# Dissection of major cancer gene variants in subsets of circulating tumor cells in advanced breast cancer

**DOI:** 10.1038/s41598-019-53660-x

**Published:** 2019-11-21

**Authors:** Stella D’Oronzo, Domenica Lovero, Raffaele Palmirotta, Luigia Stefania Stucci, Marco Tucci, Claudia Felici, Eliano Cascardi, Carmela Giardina, Paola Cafforio, Franco Silvestris

**Affiliations:** 1 Department of Biomedical Sciences and Human Oncology – Section of Internal Medicine and Clinical Oncology - University of Bari Aldo Moro, P.zza G. Cesare, 11 - 70124 Bari, Italy; 2 I.R.C.C.S-Giovanni Paolo II Cancer Institute, 70124 Bari, Italy; 3 Department of Emergency and Organs Transplant, Division of Pathology, University of Bari Aldo Moro, P.zza G. Cesare, 11 - 70124 Bari, Italy

**Keywords:** Tumour heterogeneity, Breast cancer

## Abstract

Enumeration of circulating tumor cells (CTCs) may reflect the metastatic potential of breast cancer (BC). By using the DEPArray, we investigated CTCs with respect to their epithelial-to-mesenchymal transition phenotype and compared their genomic heterogeneity with tissue biopsies. Seventeen stage IV BC patients were enrolled. Pre-enriched CTC suspensions were stained with fluorescent-labeled antibodies to epithelial (E) and mesenchymal (M) markers. CTC samples were processed by DEPArray system and clustered in relation to their markers. DNA from CTCs, as well as from primary tumor samples, was sequenced by next generation sequencing to assess the mutational state of 50 major cancer-related genes. We identified four different CTC subsets that harbored different gene variants. The most heterogenous CTC subsets included the M+/E− phenotype, which, however, expressed only 7 repeatedly mutated genes, while in the M−/E+ subset multiple mutations affected only 2 out of 50 genes. When matching all gene variants among CTC subsets, a small number of mutations was shared by only 4 genes, namely ATM, FGFR3, PIK3CA, and TP53 that, however, were absent in primary tumors. Our results postulate that the detected mutations in all CTC subsets may be considered as genomic markers of metastatic dissemination to be investigated during early stages of BC.

## Introduction

Breast cancer (BC) drives the highest incidence of cancer-related deaths in women and affects yearly more than 464,000 new patients in Europe^1^. Since the early 1970s, the survival rates have significantly improved following the introduction of novel diagnostic and therapeutic tools. The targeted therapy, indeed, definitely ameliorated the management of both early and advanced BC, although mechanisms of resistance may arise during treatment and restrain its efficacy^2^.

Besides standard diagnostic procedures, detection and quantitation of circulating tumor cells (CTCs) in the peripheral blood of BC patients are favorably accepted for prognostic purposes, for the response to treatment monitoring as well as for revealing the acquired resistance onset^3–7^. In fact, CTC count in both early and advanced BC is presently considered a prognostic criterion by the American Joint Committee on Cancer (AJCC)^8^.

Other than for quantitative analysis, CTCs have been also investigated for exploring the biology dynamics of BC by comparing their status of both hormone receptors and human epidermal growth factor receptor 2 (Her-2) with matched primary tumor samples and a variable degree of discordance was detected^9,10^. Moreover, since BC cells usually metastasize and undergo the epithelial-to-mesenchymal transition (EMT), which implies the loss of epithelial (E) morphologic and molecular pattern and the acquisition of mesenchymal (M) markers^11^, the correlation between the EMT functional status of CTCs and BC clinical outcome has been extensively investigated. In this regard, the expression of M markers emerged as an adverse prognostic factor, whereas the variations of M CTC counting during treatments, as increase or decrease, correlated with worsening of the metastatic disease or response to treatment, respectively^12–14^. In addition, the variable EMT status of CTCs was associated with different sites of metastases^15^.

This assumption raised concerns on the reliability of CTC enumeration through the Cell Search System^®^ as the Food and Drug Administration (FDA)-approved technique for CTC detection, which is based on the expression of the epithelial cell adhesion molecule (EpCAM).Thus, other methods based on the detection of additional markers, physical properties or hypermetabolic state of CTCs have been introduced^15–18^ to overcome the technical drawbacks of their fixation which prevents gene expression analyses^18–20^. To this, several Authors have recently approached the molecular characterization of CTCs to monitor cancer genomics, transcriptomics and proteomics over time, as well as correlate their variations in relation to the acquirement of drug resistance^7,21,22^. In this context, mutational analyses have been performed on CTCs through next generation sequencing (NGS) after whole genome amplification (WGA) which is a time-consuming procedure, often limited by technical and/or interpretative mistakes^23^. On the other hand, the high level of phenotypic and genetic heterogeneity that characterizes most malignancies further intrigues these analyses and their understanding^24,25^, and needs to be considered when performing molecular studies on CTCs for clinical purposes. Therefore, the genomic assessment of CTCs needs standardization of methods as well as the comparison of relative molecular variations with other genomic sources as DNA from the primary tumor.

This study was addressed at both analyzing and comparing the mutational status of CTCs, isolated from metastatic BC patients, in relation to the differential expression of EMT markers. In particular, we explored the genomic heterogeneity of CTCs by a WGA-free^23^ NGS analysis and compared the mutational status of 50 cancer-related genes in CTCs and relative primary tumors. Thus, we investigated the mutational similarities and differences existing among CTCs with different phenotypes, at both inter- and intra-patient level. Our data support the high heterogeneity of CTCs and their usefulness in investigating specific mutational derangements in relation to the EMT phenotype distribution.

## Results

### Patient and primary tumor features

Table 1 includes both demographic and major clinico-pathological features of the stage IV patients enrolled in the study, grouped in (A) and (B) as treatment-naïve or pre-treated, respectively.

**Table 1:** Demographics of treatment-naïve (A) and pre-treated (B) BC patients and major clinical-pathological features.

Clinico-pathological features							Metastatic disease						
Pt. code	Age	Time between BC diagnosis and CTC collection (months)	BC stage at diagnosis	Histological features of the primary tumor			Bone		Lung	Liver	Brain	Other sites	# of metastases (≤5; >5)
**A) Treatment-naїve patients**													
**274**	41	2	IV	Ductal, G3, Ki67 70% ER-, PgR-, Her2neu−			**+**		−	**+**	−	+	>5
**337**	49	11	IV	Ductal, G3, Ki67 54% ER+, PgR+, Her2neu−			−		**+**	−	−	+	>5
**372**	53	1	IV	Lobular, G NA, Ki67 15% ER+, PgR−, Her2neu−			**+**		−	−	−	−	>5
**382**	73	2	IV	Ductal, G3, Ki67 40% ER+, PgR+, Her2neu−			**+**		−	−	−	−	>5
**392**	77	1	IV	Ductal, G2, Ki67 18% ER+, PgR+, Her2neu−			−		**+**	−	−	+	>5
**407**	47	2	IV	Ductal, G2, Ki67 27% ER+, PgR+, Her2neu+			**+**		**+**	−	−	−	>5
**454**	65	120	III	Lobular, G3, Ki67 8% ER+, PgR+, Her2neu−			−		**+**	−	−	−	≤5
**Clinico-pathological features**								**Metastatic disease**					
**Pt code**	**Age**	**Time between BC diagnosis and CTC collection (months)**	**BC stage at diagnosis**	**Histological features of the primary tumor**	**N. of treatment lines for metastatic disease**	**Previous treatment**	**Time since last systemic treatment**	**Bone**	**Lung**	**Liver**	**Brain**	**Other sites**	**# of metastases (≤5;** >**5)**
**B) Pre-treated patients**													
**123**	54	12	III	Ductal, G3, Ki67 50% ER+, PgR+, Her2neu−	0	HT (adjuvant)	21 days	−	−	−	−	+	≤5
**242**	44	132	III	Ductal, G3, Ki67 14% ER+, PgR−, Her2neu−	8	CHT, HT	25 days	+	+	−	−	+	>5
**253**	69	260	I	Ductal, G1, Ki67 NA ER+, PgR+, Her2neu+	6	CHT, HT, TT	60 days	+	+	+	−	+	>5
**279**	49	23	IV	Ductal, G NA, Ki67 32% ER+, PgR+, Her2neu−	1	HT	21 days	+	−	−	−	−	>5
**335**	69	130	II	Ductal, G2, Ki67 36% ER+, PgR+, Her2neu−	2	CHT, HT	22 days	+	+	−	−	+	>5
**371**	56	180	III	Ductal, G2, Ki67 16% ER+, PgR+, Her2neu−	1	CHT, HT	38 days	−	−	+	−	−	≤5
**399**	61	36	III	Ductal, G3, Ki67 45% ER+, PgR+, Her2neu+	0	CHT, HT, TT (adjuvant)	28 days	−	−	−	+	−	≤5
**431**	52	132	III	Ductal, G2, Ki67 22% ER+, PgR+, Her2neu−	0	HT (adjuvant)	6 years	+	−	−	+	−	>5
**458**	55	60	III	Ductal, G3, Ki67 70% ER−, PgR−, Her2neu−	1	CHT	7 months	+	+	+	−	+	>5
**471**	67	58	III	Ductal, G3, Ki67 25% ER+, PgR+, Her2neu−	0	CHT, HT (adjuvant)	21 days	−	−	−	−	**+**	>5

Abbreviations: BC: breast cancer; NA: not available; ER: estrogen receptor; PgR: progesterone receptor; Her-2neu: human epidermal growth factor receptor 2; CHT: chemotherapy; HT: hormone treatment; TT: targeted therapy; +: yes; −: no; #: number.

As shown, BC clinical stage at the time of diagnosis ranged from I to IV. Concerning the histologic patterns, in both groups we found a variable occurrence of all tumor grading (G1-G3) as well as of Ki67 expression, whereas in addition to the variability of hormonal receptors, Her-2neu was detected in a single patient from the treatment-naïve group (A: pt. #407) and in two pre-treated patients (B: pt. #253; pt. #399).

Furthermore, with regard to the metastatic disease, at the time of CTC recruitment, all patients from both groups showed multiple metastases in different organs or at a single site. Thus, we arbitrarily defined the threshold of 5 distant metastases to discriminate the condition of oligometastatic (≤5) from plurimetastatic disease (>5). However, considering all clinico-pathological patterns, no relevant differences were observed between the two groups with respect to age, clinical stage, histopathology aspects and extent of the metastatic disease which equivalently occurred in both groups as oligo- or plurimetastatic condition. Interestingly, in a single patient (B: pt. #399) the metastatic disease included only brain metastases which also occurred in another patient (B: pt. #431) though in association to skeleton involvement.

### CTCs from BC patients show 4 EMT-related phenotypes

Spiking experiments reached sensitivity and specificity values as high as up to 98.2 ± 0.8% and 99.1 ± 0.6% respectively, with a median routing efficiency of 95% (range 80–100%), in agreement with other reports from the literature^15,26^. We obtained a recovery rate of 57.6 ± 3.8% and 55.4 ± 4.7% for MDA-MB231 and MCF-7 cells respectively, in line with previous findings by ourselves and others^23,27,28^.

**Figure 1:**
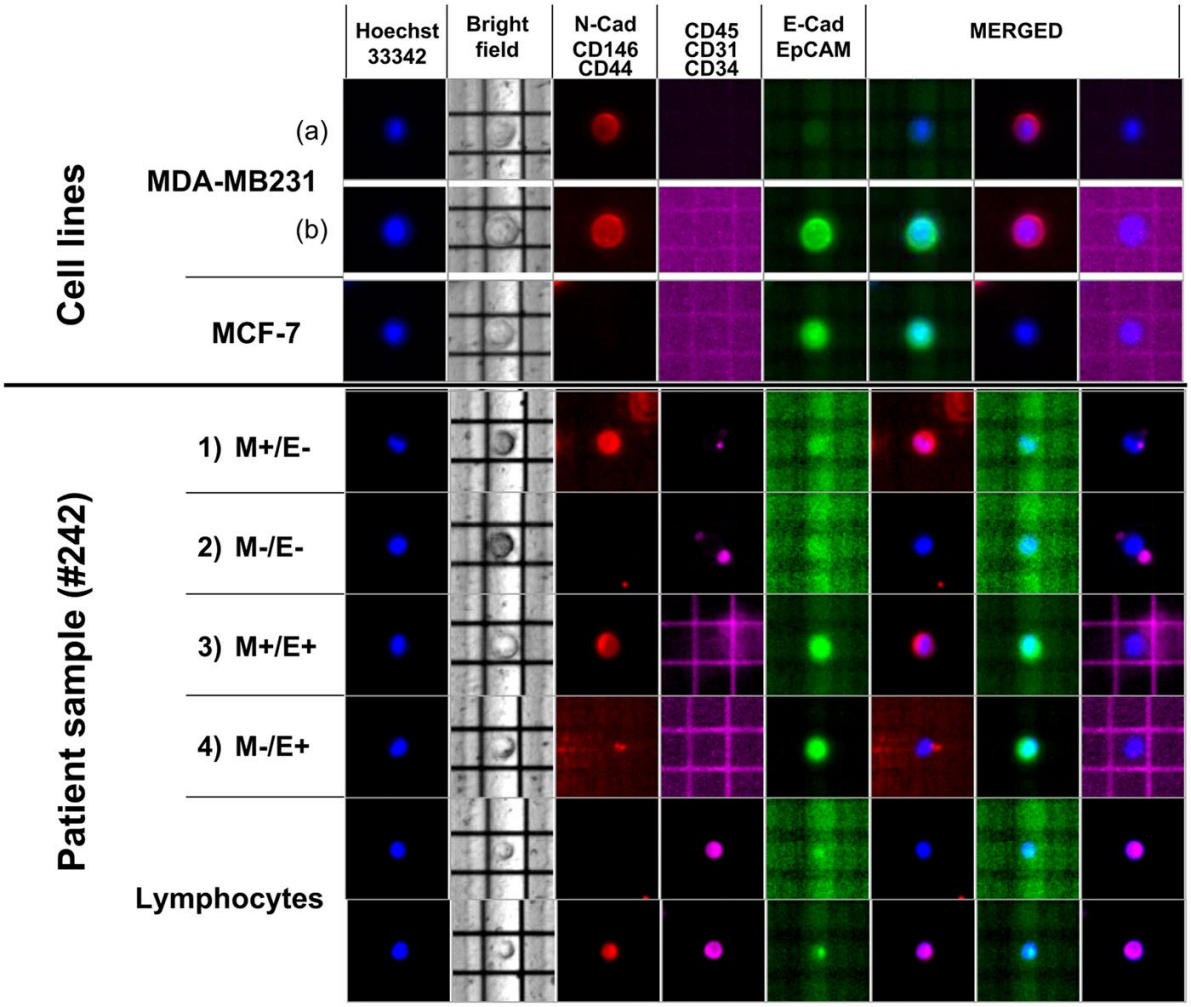
Representative images of CTCs isolated by DEPArray. The upper panel shows spiking experiments performed by using MDA-MB231 and MCF-7 BC cell lines predominantly expressing mesenchymal (M: N-cadherin/CD146/CD44; PE, red) and epithelial markers (E: E-cadherin/EPCAM; FITC, green), respectively. The lower part of the figure shows four different CTC sub-populations from the same blood sample (patient #242), showing variable expression of M and/or E markers as well as negative blood and endothelial markers (CD45/CD31/CD34; APC, purple). Nuclei are stained by Hoechst 33342 (blue). The first line shows a CTC expressing only M markers (M+/E−). Line 2 represents a CTC lacking both E and M markers (M−/E−). The APC fluorescence detectable near M−/E− CTC was due to non-nucleated blood components, namely erythrocytes and platelets that in several instances bind CTCs. Line 3 shows a CTC expressing both M and E markers (M+/E+), whereas line four depicts a CTC with E phenotype (M−/E+). Two lymphocytes derived from the CD45+ fraction of the same blood sample, are also shown as control for blood cell markers (purple), with or without CD44 expression (red).

As shown in Fig. 1, both M (N-Cad, CD146 and CD44) and E (E-Cad, and EpCAM) markers were variably expressed by both MDA-MB231 and MCF-7 BC cell lines. To support the accuracy of the method, we observed in spiking experiments that MDA-MB231 cells isolated by the DEPArray differentially showed M+/E− (a) and M+/E+ (b) phenotypes, in line with previous observation^15^. The representative fluorescence DEPArray pattern of CTCs from patient #242 also shows that the 4 phenotypes, namely M+/E−, M−/E−, M+/E+, and M−/E+, were detected in relation to the relative markers while CD45, CD31, and CD34 as hematologic and endothelial markers, were absent.

Table 2 depicts the number of CTCs clustered in different phenotypes, recruited from each patient grouped in (A) or (B). No cells with CTC-like features were isolated from the peripheral blood of healthy donors (data not shown).

**Table 2:** Number and phenotype of CTCs recovered from each patient, classified as treatment-naïve (A) or pre-treated (B), by DEPArray separation.

Patient code	M+/E−	M−/E−	M+/E+	M−/E+
**A) Treatment-naїve patients**				
274	5	0	2	0
337	69	0	14	0
372	9	0	31	5
382	33	6	5	0
392	58	2	0	0
407	57	8	12	0
454	23	6	0	0
Mean value	**36.28**	**3.14**	**9.14**	**0.71**
Median value	**33**	**2**	**5**	**0**
**B) Pre-treated patients**				
123	3	0	4	0
242	9	6	9	2
253	0	20	39	8
279	22	14	12	0
335	43	0	0	14
371	6	3	0	0
399	42	34	0	0
431	60	46	0	0
458	6	6	0	0
471	29	13	8	0
Mean value	**22**	**14.2**	**7.2**	**2.4**
Median value	**15.5**	**9.5**	**2**	**0**
Total	**474**	**164**	**136**	**29**
Median value	**23**	**6**	**4**	**0**

Abbreviations: M+/E−: CTCs expressing only mesenchymal markers; M−/E−: CTCs which do not express either mesenchymal or epithelial markers; M+/E+: CTCs expressing both mesenchymal and epithelial markers; M−/E+: CTCs expressing only epithelial markers.

Based on fluorescence analysis by Cell Browser Software, recruited CTCs were clustered as M+/E−, M−/E−, M+/E+, and M−/E+ subsets, as depicted in Fig. 1. In particular, besides CTCs expressing E and/or M markers, we identified a subpopulation of circulating cells which met all the criteria for CTC identification (e.g. round or oval cell shape, positive DAPI staining, nuclear integrity, negative staining for CD45, CD31 and CD34)^26,29,30^ but exhibited absent or weak expression of both phenotype markers, as reported by others^27,29,31^; thus we defined these cells as double negative (M−/E−) CTCs.

Analysis of quantitative data revealed that, although in the presence of small groups of patients, both mean and median values of the absolute number of several CTC subsets were variable between the two groups. In particular, the M+/E− CTCs were numerically more represented in group (A) in contrast with M−/E− CTCs whose values were higher in patients of group (B) (p < 0.0001). Conversely, no significant differences were observed between the two groups in the remaining CTC subsets (p > 0.05).

**Figure 2:**
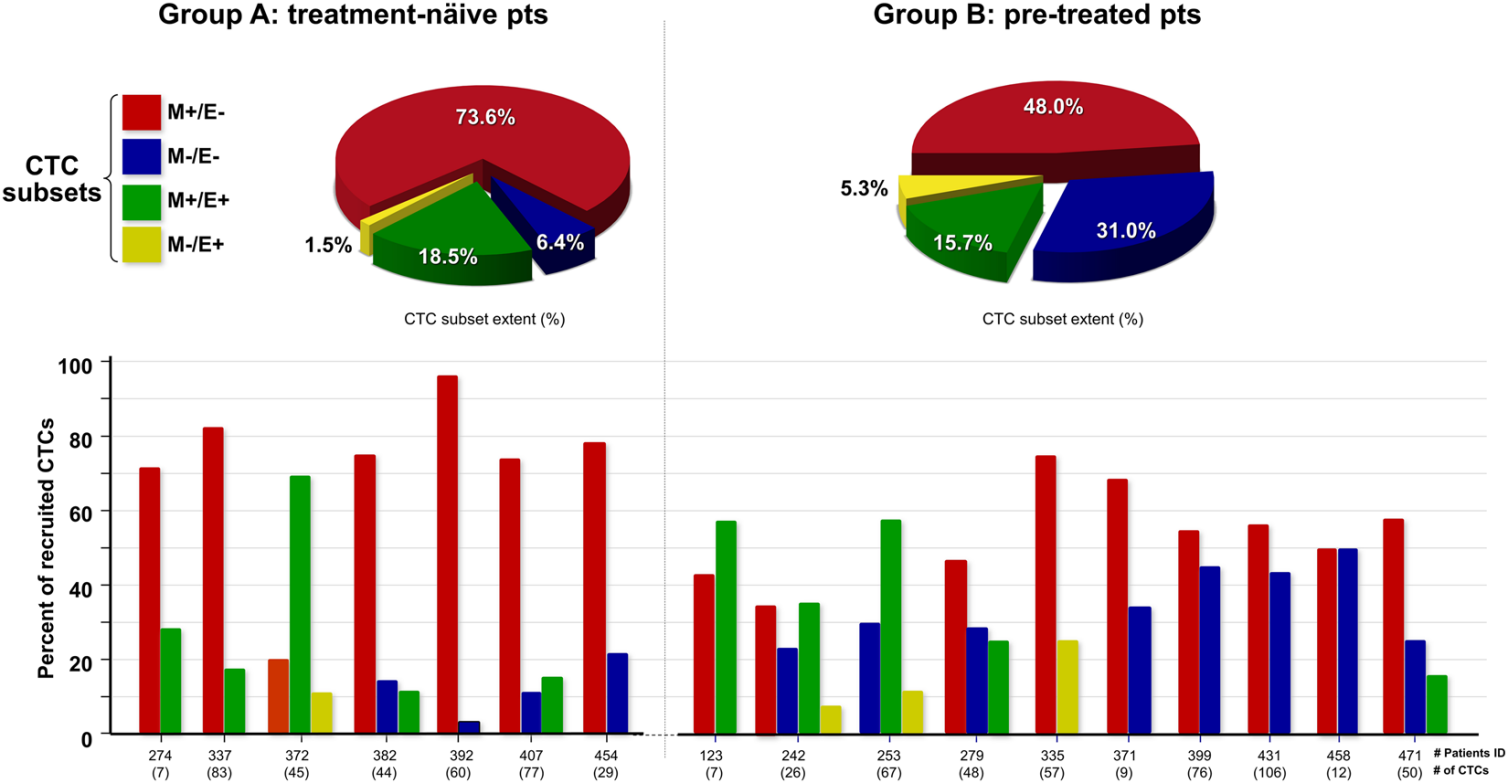
Distribution of CTC subsets. Magnitude of EMT-related CTCs from metastatic BC patients, grouped as A (treatment-naïve) or B (pre-treated). The upper section depicts percent values of these cohorts with significant differences regarding both M+/E− and M−/E− subsets. The lower part shows the extent of all CTC subsets in each patient depicting intra- and inter-patient heterogeneity, even within the same group of patients. The numbers in brackets are the recovered CTCs from each patient (range: 7–106). Abbreviations: CTCs: circulating tumor cells; M+/E−: CTCs expressing only M markers; M−/E−: CTCs negative for both M and E markers; M+/E+: CTCs expressing both M and E markers; M−/E+: CTCs expressing only E markers.

A representative subset distribution of the CTCs within each group (up) and in single patients (down) is depicted in Fig. 2. As shown in the upper section, the magnitudes of both M+/E− and M−/E− CTCs, as percentage of subset expansion, were visibly different between both groups [M+/E−: 73.6% in (A) vs 48.0% in (B); M−/E−: 6.4% in (A) vs 31.0% in (B)] in contrast with the M+/E+ and M−/E+ CTC distribution. The extent of the CTC subsets in each patient also reflected variable values (lower section). We observed individual variability of all four phenotypes in both groups of patients without a clear association with defined clinical patterns of the disease. For instance, although the M+/E− phenotype was predominant in group (A), its association with a single additional subset occurred globally in 10 patients (A: pts #274; #337; #392; #454; B: pts #123; #335; #371; #399; #431; #458) and within this subgroup, the association of M+/E− with M–/E– phenotypes was prevalent (6 pts: #392; #454;#371; #399; #431; #458). On the other hand, a single heavily pre-treated patient (pt. #242) expressed all four subsets as potential significance of clonal heterogeneity following multiple anti-cancer treatments^25^.

These results provided evidence of the high EMT-related phenotype heterogeneity of CTCs isolated from both treatment-naïve and pre-treated BC patients. However, the M+/E− subset appeared the most recurrent in both groups of patients although its extent was higher in those who were never treated before. By contrast, the double-negative subset (M−/E−) significantly occurred in pre-treated patients and in single instances, as in pts #399 and #431,was associated with the formation of brain metastases as major clinical sign of tumor progression.

### CTCs from BC patients are molecularly heterogeneous by NGS analysis of major cancer related genes

The next set of experiments was addressed to reveal and compare both pathogenic and non-pathogenic variants in DNA from CTCs and relative tumor formalin-fixed paraffin-embedded (FFPE) samples. Table 3 describes the mutations detected on the available FFPE specimens and on CTC subsets in each patient. As shown, in relation to the available substrates, we detected a number of pathogenic mutations in PIK3CA, TP53, ATM and PTEN genes in 7 FPPE samples, while the remaining 4 specimens harbored no pathogenic variants (pt. #337, pt. #392, pt. #407, and pt. #454). In addition, in the FFPE sample from pt. #123, two pathogenic variants were detected for PIK3CA and one for ATM. In two patients (#242 and #471) the concentration of FFPE-derived DNA was 1 ng/µl and the yield of barcoded libraries after quantification was much less than 100 pM. Hence, these samples were inadequate for template preparation and for the subsequent steps of mutational analysis. We interpreted that such a few recruited DNA amount was probably an effect of neo-adjuvant treatment administered to both patients.

**Table 3:** Pathogenic gene variants identified by targeted Next Generation Sequencing performed by using the Ion AmpliSeq™ Cancer Hotspot Panel v2.

Patient code	FFPE	M+/E−	M−/E−	M+/E+	M−/E+
**A) Treatment naїve patients**					
274	TP53 c.833 C > T p.P278L	No pathogenic variants identified	Not available	No pathogenic variants identified	Not available
337	No pathogenic variants identified	AKT c.528 C > A p.Tyr176* ATM c.3878 A > G p.N1293S BRAF c.1379 G > T p.Gly460Val KIT c.1904A > G p.E635G **PIK3CA c.3196 G > A p.A1066T** NOTCH1 c.5026 G > A p.V1676I	Not available	ALK c.3836 + 1 G > TSplice site NRAS c.394 G > Tp.E132Ter PIK3CA c.352 + 1 G > A Splice site PDGFRA c.1955G > Tp.G652V **PIK3CA c.3196 G > A p.A1066T**	Not available
372	Not available	SMO c.1180 T > C p.T394H KRAS c.153 T > C p.C51C **TP53 c.388 C > A p.L130I** **PIK3CA c.3140 A > G p.H1047R** TP53 c.667 C > T p.P223S FBXW7 c.1442 C > T p.A481V GNAQ c.735 + 1 G > T Splice site MET c.1124 A > G p.N375S	Not available	**ATM c.1810C > T p.P604S** **FGFR3 c.1150 T > C p.F384L** **PIK3CA c.3140 A > G p.H1047R** TP53 c.742 C > T p.R248TW	**ATM c.1810C > T p.P604S** **FGFR3 c.1150 T > C p.F384L** **TP53 c.388 C > A p.L130I** KRAS c.103 A > G p.T35A SMO c.1870A > T p.Lys624Ter RB1 c.1975T > C p.Y659H EGFR c.2300 C > T p.A767V JAK3 c.2126 G > A p.Trp709Ter
382	Not available	**PIK3CA c.3196 G > Ap.A1066T** **PIK3CA c.3140 A > G H1047R** **FGFR3 c.1150 T > C p.F384L**	**FGFR3 c.1150 T > C p.F384**	**PIK3CA c.3196 G > Ap.A1066T** **PIK3CA c.3140 A > G H1047R** TP53 c.1009 C > T p.R337C	Not available
392	No pathogenic variants identified	ERBB4 c.866 G > T p.C289F PIK3CA c.1633G > A p.E545K	No pathogenic variants identified	Not available	Not available
407	No pathogenic variants identified	PTEN c.1001 A > G p.N334S PTEN c.511 C > T p.Gln171Ter TP53 c.388 C > A p.L130I	No pathogenic variants identified	PIK3CAc.3140 A > T p.H1047L	Not available
454	No pathogenic variants identified	TP53 c.388 C > A p.L130I	No pathogenic variants identified	Not available	Not available
**B) Pre-treated patients**					
123	**ATM c.7328 G > A p.R2443Q** PIK3CA c.1624G > C p.E542Q PIK3CA c.3145 G > C p.G1049R	**ATM c.7328 G > A p.R2443Q** SMAD4 c.512 C > A p.Ser171*	Not available	No pathogenic variants identified	Not available
242	Inadequate sample	**TP53 c.388 C > A p.L130I** PIK3CA c.323 G > A p.R108H SMAD4 c.1216 G > A p.A406T STK11 c.536 C > T p.P179L	No pathogenic variants identified	ATM c.1810C > T p.P604S	**TP53 c.388 C > A p.L130I** NRAS c.181 C > A p.Q61K
253	Not available	Not available	**PIK3CA c.1633G > A p.E545K** **PIK3CA c.3140 A > T p.H1047L** **TP53 c.742 C > T p.R248TW**	FBXW7 c.1451 G > T p.R484M SMO c.646 C > Tp.Q216Ter	**PIK3CA** **c.1633G > A p.E545K** **PIK3CA** **c.3140 A > T p.H1047L** **TP53** **c.742 C > T p.R248TW**
279	PIK3CA c.3140 A > T p.H1047L	**ATM c.1810C > T p.P604S** FGFR3 c.1150 T > C p.F384L	**ATM c.1810C > T p.P604S** EGFR c.2257 C > Tp.P753S	FGFR3 c.2408 G > Ap.G803D HNF1A c.620 G > A p.G207D	Not available
335	PTEN c.959 T > A p.L320*	PTEN c.959 T > A p.L320* APC c.4348 C > T p.R1450* ATM c.7328 G > A p.R2443Q KDR c.2946 C > T p.S982S MET c.2962 C > T p.R988C	Not available	Not available	Inadequate sample
371	PIK3CA c.1633G > Ap.E545K	ATM c.1810C > T p.P604S PTEN c.991 G > A p.D331N PTEN c.635-1 G > A p.? PTPN11 c.169 C > T p.Q57*	TP53 c.388 C > A p.L130I	Not available	Not available
399	TP53 c.711 G > A p.M237I	No pathogenic variants identified	No pathogenic variants identified	Not available	Not available
431	Not available	No pathogenic variants identified	ATM c.5415 G > A p.W1805* BRAF c.1800G > A p.V600V FGFR3 c.2115 G > A p.K705K RET c.2691 A > G p.R897R	Not available	Not available
458	TP53 c.817 C > T p.R273C	No pathogenic variants identified	No pathogenic variants identified	Not available	Not available
471	Inadequate sample	No pathogenic variants identified	**TP53 c.388 C > A p.L130I**	**TP53 c.388 C > A p.L130I** CDKN2A c.241 C > T p.P81S CTNNB1 c.136 C > T p.L46L KDR c.2959 G > T p.E987Ter TP53 c.713 G > A p.C238Y	Not available

Abbreviations: FFPE: formalin-fixed paraffin-embedded (FFPE) primary breast cancer samples; M+/E–: CTCs expressing mesenchymal markers only; M−/E−: CTCs which do not express either mesenchymal or epithelial markers; M+/E+: CTCs expressing both mesenchymal and epithelial markers; M−/E+: CTCs expressing epithelial markers only.

Targeted NGS analysis on genomic DNA, extracted from patients’ white blood cells (WBC), was performed to exclude all germline sequence variants during the evaluation of mutational results, as previously described^32,33^. As shown in Supplementary Tables, we found several discrepancies at both intra-patient and inter-patient levels and, although in few instances we found shared gene variants in primary tumors matched with CTC samples (e.g. ATM*c.7328G* > *A* in both FFPE sample and M+/E− CTCs from pt. #123, whereas the PTEN *c.959* *T* > *A* variant was similarly revealed in both FFPE and M+/E− CTCs from pt. #335), the majority of pathogenic variants detected in CTCs were not revealed in the corresponding primary tumor (Table 3). This has been also described in previous studies^34,35^.

Interestingly, several gene variants co-existed in different CTC subsets though undetected in relative primary tumors (i.e.: pt. #337, PIK3CA c.3196G > A; pt. #372, ATM c.1810C > T, FGFR3 c.1150T > C, TP53 c.388C > A, PIK3CA c.3140A > G; pt. #471, TP53 c.388C > A). This aspect was interpreted as an effect of the achievement of novel mutations by CTCs, potentially conferring selective advantages in terms of survival and metastatic potential^26^. This assumption is also supported by the observation that, in comparison with matched FFPE tumor samples, CTCs usually harbor more gene variants that support their elevated genomic instability. Table 4 includes the total numbers of both pathogenic and non-pathogenic variants in all substrates from patients, once again grouped in (A) and (B). As can be seen, the highest numbers of gene variants occurred in the M+/E− CTC subsets from both groups of patients. However, while the global number of either pathogenic or non-pathogenic variants was slightly variable between the two groups (pathogenic variants A = 46; B = 51; non-pathogenic variants: A = 177; B = 227), the higher numbers of non-pathogenic gene variants were putatively ascribed to genomic activation of survival mechanisms once CTCs enter the bloodstream, as postulated^36,37^, and this apparently occurred in both groups of our patients.

**Table 4:** Number of pathogenic and non-pathogenic gene variants identified in FFPE samples and in single CTC suspensions from each patient.

Patient code	Pathogenic Variants					Tot.	Non-Pathogenic Variants					Tot.
	FFPE	M+/E−	M−/E−	M+/E+	M−/E+		FFPE	M+/E−	M−/E−	M+/E+	M−/E+	
**A) Treatment naїve patients**												
274	1	0	—	0	—	1	—	1	—	0	—	1
337	0	6	—	5	—	11	—	20	—	18	—	38
372	—	8	—	4	8	20	—	33	—	4	39	76
382	—	3	1	3	—	7	—	5	0	10	—	15
392	0	2	0	—	—	2	—	19	0	—	—	19
407	0	3	0	1	—	4	0	16	1	9	—	26
454	0	1	0	—	—	1	—	0	2	—	—	2
Total	1	23	1	13	8	46	0	94	3	41	39	177
**B) Pre-treated patients**												
123	3	2	—	0	—	5	1	38	—	31	—	70
242	—	4	0	1	2	7	—	31	1	1	17	50
253	—	—	3	2	3	8	—	—	0	13	0	13
279	1	2	2	2	—	7	—	11	6	1	—	18
335	1	5	—	—	—	6	3	16	—	—	—	19
371	1	4	1	—	—	6	—	12	6	—	—	18
399	1	0	0	—	—	1	—	0	1	—	—	1
431	—	0	4	—	—	4	—	9	10	—	—	19
458	1	0	0	—	—	1	—	0	1	—	—	1
471	—	0	1	5	—	6	—	3	4	11	—	18
Total	8	17	11	10	5	51	4	120	29	57	17	227
Total (A + B)	9	40	12	23	13		4	214	32	98	56	

Abbreviations: FFPE: formalin-fixed paraffin-embedded (FFPE) primary breast cancer samples; M+/E–: CTCs expressing mesenchymal markers only; M−/E−: CTCs which do not express either mesenchymal or epithelial markers; M+/E+: CTCs expressing both mesenchymal and epithelial markers; M−/E+: CTCs expressing epithelial markers only.

### CTC subsets are inter- and intra-patient genomically heterogeneous

As shown in Table 2, we collected at least two different CTC sub-populations from all patients. Thus, with respect to the sample extent, we analyzed cell suspensions of 2 to 5 CTCs. Results are reported in Supplementary Tables which show the high heterogeneity both at intra- and inter-patient levels. However, in most patients we identified one or more gene variants which were constantly shared among parental CTC subpopulations. For instance, in CTCs isolated from pt. #242, a non-pathogenic variant (SMAD4 c.1335A > G) was detected in all CTC subsets, although at variable allele frequency (range 5.2–14.0%). Similarly, all CTC subsets from pt. #372 shared several variants (ERBB4 c.421 + 58 A > G; KDR c.798 + 54G > A; PDGFRA c.2472C > T; STK11 c.465-51T > C). In other patients from both groups (i.e. pts #123, 253, 279, 337, 371, 382, 407, 471) we observed at least two CTC subsets harboring the same gene variants thus supporting the spreading in blood of different tumor cell sub-clones still retaining molecular traces of their common origin.

**Figure 3:**
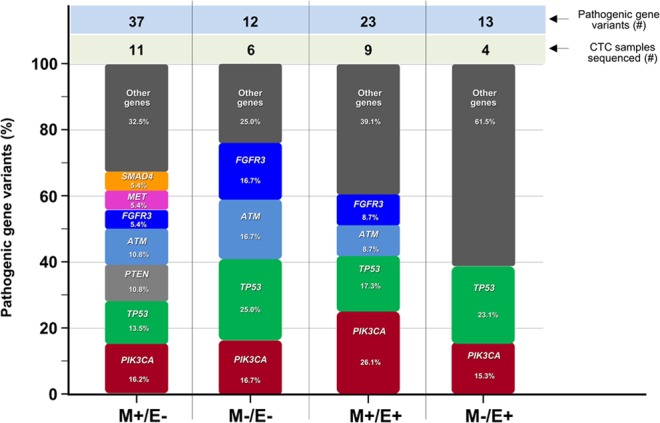
Percent of pathogenic variants recurring in single genes in EMT-related CTC subsets. Genes showing a unique variant were included in ‘other genes’ box. By contrast, the recurrence of multiple variants in single genes is expressed as percent values. As shown, a restricted number of genes, namely 7 in M+/E− and only 2 in M−/E+ subset, out of 50 genes of the Cancer Hotspot panel, harbored repeated mutations by NGS. The most mutated genes in all CTC subsets included PIK3CA, TP53, FGFR3, and ATM, whereas PTEN also expressed multiple variants although only in the M+/E− CTCs to further support high genomic heterogeneity in this subset. Numbers refer to variants and sequenced CTCs. Abbreviations: CTCs: circulating tumor cells; M+/E−: CTCs expressing mesenchymal markers only; M−/E−: CTCs which do not express either mesenchymal or epithelial markers; M+/E+: CTCs expressing both mesenchymal and epithelial markers; M−/E+: CTCs expressing epithelial markers only; #: number.

Figure 3 depicts in each CTC subset the percentage of pathogenic variants recurring in single genes with respect to the full number of pathogenic variants. Genes showing a unique variant were grouped and indicated as ‘other genes’. As shown, a restricted number of genes, namely up to 7 in M+/E− subset and only 2 in M−/E+ subset out of 50 genes of the Cancer Hotspot panel, harbored major numbers of mutations. In fact, the M+/E− CTCs showed the highest number of mutated genes involving PIK3CA at 16.2% of all mutations, TP53 at 13.5%, PTEN and ATM at 10.8%, FGFR3, MET and SMAD4 at 5.4%. By contrast, the other subsets showed a minor number of repeatedly mutated genes, namely 4 in M−/E−, 4 in M+/E+ and 2 in M−/E+ subset. This analysis suggested that major cancer related genes as TP53 and PIK3CA harbored the highest number of mutations in the overall CTC population, followed by FGFR3 and ATM, whereas the M−/E+ subset included only TP53 and PIK3CA as main mutated genes. Of interest, TP53 (25%), FGFR3 (16.7%) and ATM (16.7%) were predominantly mutated in M−/E− CTCs with respect to the other subsets.

To support the highest heterogeneity of the M+/E− CTC subset, Table 4 shows that the majority of both pathogenic and non-pathogenic gene variants recur in this subpopulation, suggesting a potential correlation between the higher genomic instability of these cells and their survival in the bloodstream^34,35,38,39^.

With regard to the correlation of mutational assessment with the extent of the metastatic disease, we observed a higher mean number of pathogenic CTC mutations/patient in women with high compared to those with low tumor burden (6.07 vs 2.00). However, this difference was not statistically significant (p > 0.05) although reflecting the general opinion that the tumor burden may linearly correlate with higher genomic instability.

**Figure 4:**
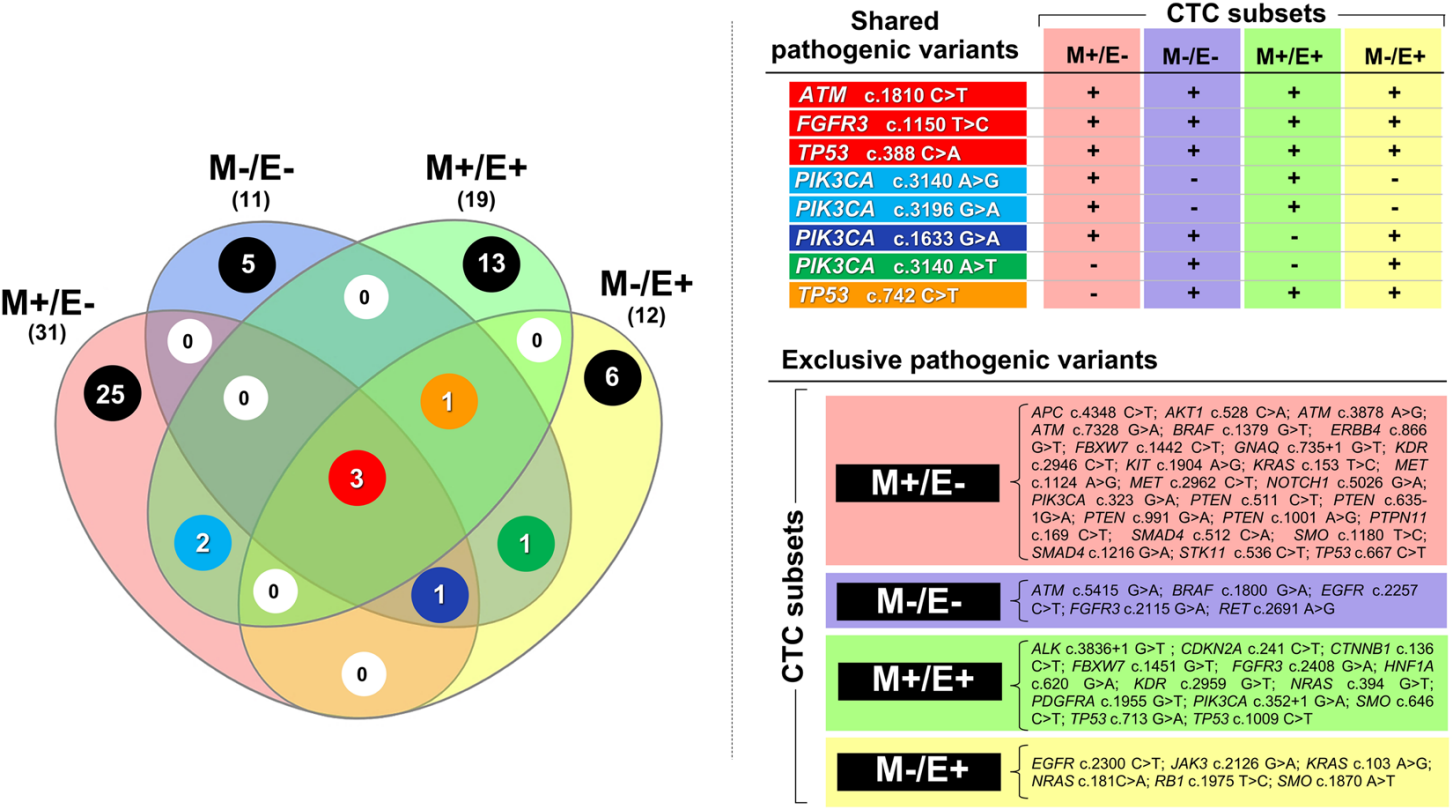
Venn diagram depicting both shared and exclusive pathogenic variants in CTC subsets. Numbers of the variants shared between the subsets are differently colored while those of the exclusive mutations are highlighted in black (left). Shared variants involved only 4 genes (ATM, FGFR3, TP53, and PIK3CA), whereas the subset-specific mutations were at higher number of genes in each CTC subpopulation (right).

Finally, since the 4 CTC subsets were originally derived from primary tumors and expressed variable mutational status, as described in Table 3, we explored the recurrence of mutations within all CTC subsets. Figure 4 depicts a Venn diagram (left) including the number of exclusive and shared mutations by all CTC subsets. We found that 8 gene variants were variably shared by all subsets. In fact, as depicted, 3 gene variants (red: ATM c.1810 C > T; FGFR3 c.1150 T > C; TP53 c.388 C > A) occurred in all subsets, whereas 2 mutations (blue: PIK3CA c.3140 A > G; PIK3CA c.3196 G > A) were shared by 2 CTC subsets, a single one (orange: TP53 c.742 C > T) in 3 subsets, another one (dark blue: PIK3CA c.1633 G > A) in 3 subsets, and the last single variant (green: PIK3CA c.3140 A > T) in 2 CTC subsets. On the contrary, phenotype-exclusive mutations are indicated in black and numerically more represented in M+/E− subset. The right section of the figure lists all shared and exclusive variants detected in all CTC subsets.

This analysis suggested that at least 3 mutations recurred in all CTC subsets from all patients and that the presence of such a restricted number of pathogenic gene variants reflects the common tumor clonal origin, while the highest number of exclusive and shared mutations in M+/E− subset may support the clonal evolution of this CTC subpopulation to allow the tumor progression.

## Discussion

Over the last decade, several Authors approached CTC isolation from patients with early and advanced BC primarily for prognostic purposes as well as for monitoring the treatment efficacy and the disease progression, including the rise of acquired resistance^3–6^. To date, the only FDA-approved technology for CTC enumeration is the Cell Search® System, whose capability to isolate CTCs relies on the selective identification of EpCAM on these cells^40^. However, recent evidence suggests that a variable proportion of EpCAM-negative BC cells can also be detected in the peripheral blood of patients, in relation to the expression of EMT markers, thus providing more information for metastatic evolution and management of the disease^12–15,21,41^.

To this regard, several investigators have recently described a variable mutational status of PIK3CA between EpCAM^high^ and EpCAM^low/negative^ CTCs isolated from BC patients which was apparently associated to a different clinical evolution^29^. This suggests that phenotypical differences also correlate with the molecular heterogeneity of cancer cell sub-clones and that these associations need to be intensively investigated when performing mutational analyses on CTCs for clinical purposes as the detection of biomarkers for treatment options or therapy resistance which are recently emerging in BC setting^22–24^.

In our study, a DEPArray-based strategy was employed to detect and isolate viable CTCs from the peripheral blood of advanced BC patients in relation to the expression of EMT markers, and in line with data from Bulfoni and co-workers^15^, we selected four CTC sub-populations that could be easily distinguishable by the expression of E and/or M markers, or the absence of both. Indeed, while several Authors have theorized that EMT is fragmented in several stages^11,42,43^, we focused on the most representative sub-populations, in agreement with previous reports^14,15^.

In agreement with previous findings^15^, the M+/E− subset accounted for the majority of isolated CTCs in both groups of treatment-naïve and pre-treated patients. However, in the treatment-naïve group the percent distribution of this CTC fraction was more consistent than in pre-treated patients in contrast with the M−/E− subset whose extent was apparently higher in these patients. Although this CTC subset has been associated to the occurrence of brain metastases^15^, we interpreted this discrepancy as an effect of multiple treatments received by these patients which probably reverted the EMT marker expression in a subpopulation of CTCs. On the other hand, we detected all 4 CTC subtypes only in a single heavily pre-treated patient (#242) with extensive metastatic disease, and we interpreted the wide phenotype heterogeneity of CTCs as probably dependent on the previous multiple treatments.

With the purpose to explore further differences between CTC fractions in BC, we next explored the mutational status of 50 oncogenes and tumor suppressor genes and compared data from CTCs with matched primary tumor samples. We found high intra-patient and inter-patient genomic heterogeneity, as reported by others^34,35^. In particular, we observed that most gene variants detected in CTCs, even if shared among CTC sub-populations, were not detectable in corresponding FFPE samples, suggesting that the acquisition of specific mutations could confer to the spread cells a proliferative and survival advantage over other sub-clones within the primary tumor.

When focusing on CTC sub-populations, we still found high heterogeneity, especially in terms of pathogenic gene variants which were mainly “phenotype-specific”. Remarkably, the highest number of either pathogenic or non-pathogenic variants was detected in M+/E− cells, namely the most recurrent subset within the entire population of CTCs (Supplementary Tables), thus supporting the hypothesis that genomic instability of cancer cells may contribute to their survival within and outside the primary malignancy. Coherently, we found a higher mean number of pathogenic gene variants in the CTC samples of patients with more than 5 distant metastases, as compared to those with less extensive disease. On the other hand, the increased number of non-pathogenic variants in pre-treated patients reflected the additional mutational evolution of CTCs to survive and probably escape suppressor mechanisms.

It has been reported that both phenotypic and genomic heterogeneity of CTCs may reflect the different mechanisms underlying their entrance in the bloodstream. To this regard, several Authors hypothesize that CTCs are capable of either passive or active intravasation, due to cell shedding which is induced by mechanical forces or migration mechanisms enhanced by the EMT process^36,37^. In line with these observations, in our work we found a wide inter- and intra-patient variability both in the extent of CTC fractions and in gene variants.

Several Authors also described such heterogeneity at single-CTC level^26,29,33–35,44^, but the minimal amount of DNA extracted from a single cell requires preliminary WGA for downstream molecular analyses. WGA is a time-consuming procedure that may provide technical errors such as inadequate coverage, allelic dropouts, false negative and false positive results^23,38^ that may affect the analytical quality of NGS procedures^18,38^. Hence, based on our previous data^23^, we applied a WGA-free targeted NGS analysis for the mutational comparison of small CTC numbers (2 to 5 cells). Other Authors suggest that the accuracy of molecular investigation on CTCs might be improved by increasing the number of analyzed cells^7,45–49^. However, data from our WGA-free NGS on such a low number of CTC subsets supports the validity of our method and once again confirms the wide genomic heterogeneity occurring in all samples and particularly in the M+/E− subset.

A striking result from any analysis of our data concerns the correlation of EMT phenotype of CTCs and mutational screening from targeted NGS of approximately 2,800 COSMIC mutations in selected hotspot regions. We found that within the 50 investigated genes, a restricted number of genes is variably and repeatedly mutated in different CTC subsets. The highest number of genes was 7 in M+/E− subpopulations in contrast with only 2 in the M−/E+ subset, thus suggesting that such a limited number of cancer related genes recur at variable mutated status in all subsets and that, once again, the M+/E− subset maintains its higher heterogeneity with respect to the other subpopulations. A further contribution to this interpretation was also provided by the Venn diagram of shared gene variants among the CTC subsets. Three variants affecting ATM, FGFR3 and TP53 recur in all CTCs and, in addition to other shared mutations, suggest the common deregulation of key genes in advanced BC.

With respect to FGFR3 mutations, it is worth to mention that the International Cancer Genome Consortium (ICGC Data Portal https://dcc.icgc.org) indicates relatively low frequencies of such variants in breast malignancies. Interestingly, we identified FGFR3 gene mutations in CTCs and not in matched primary tumors from 4 patients, which is in agreement with previous studies reporting ex-novo FGFR3 mutations in CTCs from BC patients^35,50^ as well as in metastatic BC samples, compared to primary tumors^51,52^.

As depicted in Fig. 4, a total number of 4 genes, namely ATM, FGFR3, TP53, and PIK3CA, are repeatedly mutated in all CTCs from our study and, considering that in all instances we evaluated BC patients with tumor progression and metastatic disease, we postulate that the expression of those major gene variants in CTCs could reflect their propensity to the metastatic activity. However, due to the limited number of genes analyzed, we cannot exclude the presence of other genomic alterations, for which further whole genome sequencing is necessary.

## Methods

### Patients

Seventeen female BC patients with metastatic disease, hospitalized at the Medical Oncology Unit of the University Hospital “Policlinico of Bari”, were enrolled after written informed consent. The protocol was approved by the Ethics Committee of the University of Bari (Project identification code: 44100). The study was performed in accordance with the principles of the Declaration of Helsinki; clinical data were collected from all patients and anonymized. The eligibility criteria for patient recruitment were: adult (≥18 years) female patients with metastatic BC who were systemic-treatment naïve, or with clinical and/or radiological evidence of disease progression during systemic therapy for metastatic disease. The patients were enrolled at least 21 days after the last cycle of treatment. Personal history of other synchronous or metachronous malignancies represented an exclusion criterion. In parallel, a FFPE histology sample of primary tumors from the same patients was recovered from the Pathology Division of the University of Bari for comparative mutational analyses.

### Cell lines, cultures and spiking experiments

To characterize EMT-related phenotypes, both MCF-7 and MDA-MB-231 human BC cell lines (ATCC, Manassas, Virginia, USA) were used for preliminary experiments exploring their differential expression of both E and M markers^15^. Cells were cultured in complete RPMI 1640 medium (10% fetal bovine serum plus 1% penicillin-streptomicin; Gibco®, Waltham, MA, USA) and grown at 37 °C in a 5% CO_2_ incubator.

Both BC cell lines at the density of 1 × 10^3^ cells were spiked into 15 ml of healthy donor peripheral blood, as described^15^, to determine the sensibility of the procedure and the tumor cell recovery rate. Spiking experiments were performed in triplicate by using both BC cell lines. Thus, the cell samples were centrifuged through Ficoll Histopaque (Sigma-Aldrich, Milan, Italy) density gradient and the cell suspensions were enriched in tumor cells by immunomagnetic negative selection in the AutoMACS separator (Miltenyi Biotech, Bergisch Gladbach, Germany) using anti-CD45 and anti-glycophorin monoclonal antibodies (MoAbs) conjugated with magnetic microbeads (Miltenyi) to exclude both peripheral blood mononuclear cells (PBMCs) and erythrocytes.

The tumor cell enriched suspensions were then incubated with a mixture of fluorescent-labeled MoAbs to E or M markers, as described^15^, namely fluorescein isothiocyanate (FITC)-conjugated anti-EpCAM (Becton Dickinson, San Jose, CA, USA) and anti-E-cadherin (BD Pharmingen, San Diego, CA, USA) for E markers, and phycoerythrin (PE)-conjugated anti-CD44 (BioLegend, San Diego, CA, USA), anti-CD146 (BD Pharmingen), and anti-N-cadherin (BD Pharmingen) MoAbs for the M phenotype. Furthermore, anti-CD45, anti-CD31, and anti-CD34 (Thermo Fisher Scientific) allophycocyanin (APC)-conjugated MoAbs were used to discriminate residual blood and endothelial cells. Nuclei of viable cells were stained by using Hoechst 33342 (Sigma-Aldrich).

Labeled samples were loaded into an A300K DS V2.0 cartridge and processed by the DEPArray dielectrophoretic system (Menarini, Silicon Biosystem, Castel Maggiore, Italy). In this equipment cell samples were thus scanned under an automated fluorescence microscope to generate image gallery through the Cell Browser Software^23^ to provide positive controls for the detection of both E and M markers on CTCs.

### CTC detection and isolation

Fifteen ml of peripheral blood from each patient were collected after discarding the first 5 ml to avoid contamination with E skin cells. After gradient stratification on Ficoll Histopaque (Sigma-Aldrich) and immunomagnetic negative selection, the samples were stained with the described MoAb mixture. CTCs were sorted by DEPArray and selected in relation to cell shape (round or oval), positive DAPI staining, nuclear integrity, negative staining for CD45, CD31 and CD34^26,29,30^, as well as cell diameter comprised between 7 and 40 µm, according to the Technical Specification of DEPArray V 2.0 User Manual.

CTCs were thus grouped according to the expression of E and/or M surface markers or their absence, then moved through dielectrophoretic cages in the parking camera. Finally, the cells were recovered in 0.2 ml Eppendorf (Hamburg, Germany) tubes and processed for volume reduction in phosphate buffered saline before molecular analyses. Residual lymphocytes in CTC-enriched samples represented the negative control for E and M markers.

### Targeted NGS analysis

DNA from 10 µm FFPE primary tumor sections as well as from WBC was extracted by QIAamp DNA FFPE Tissue Kit (Qiagen), and DNeasy® Blood & Tissue Kit (Qiagen, Hilden, Germany) respectively^53,54^, and then quantified by Qubit® 3.0 fluorometer (Life Technologies™ Carlsbad, CA, USA). For mutational analyses, Ion AmpliSeq™ Cancer Hotspot Panel v2 (Life Technologies), a commercial kit detecting 2,800 somatic mutations in 50 cancer-associated genes including both oncogenes and tumor suppressor genes, was employed as reported^23^. Briefly, 10 ng of DNA were used to construct the barcoded libraries through both Ion AmpliSeq™ Library kit 2.0 and Ion Xpress™ barcode adapters (Life Technologies). The quality and quantity of libraries, purified with Agentcourt AMPure XP (Beckman Coulter, Indianapolis, USA), were evaluated by the Ion Library TaqMan Quantitation Kit (Life Technologies) on the StepOne Plus system (Applied Biosystem, Foster City, California, USA). Finally, libraries were templated with the Ion OneTouch™ 2 System and Ion OneTouch™ ES, and then sequenced on the NGS Ion Torrent PGM™ system by using Ion Torrent™ 316 or 318 chips.

For CTC mutational analysis, we applied our recently described protocol, based on direct sequence analysis of CTCs without the pre-analytical steps of DNA extraction and WGA^23^. Briefly, multiple CTC pools including 2 to 5 cells depending on sample cell content, and phenotype-clustered were selected for each patient and lysed with the Lysis Reaction Mix (Menarini Silicon Biosystems). The subsequent barcoded libraries were obtained by increasing from 18 to 25 the number of cycles indicated in the “Amplify the Targets” section of the Ion AmpliSeq™ DNA Library preparation user guide (Ion AmpliSeq™ Library Preparation, Quick Reference, Publication Number MAN0006735 Revision E.0).Verification of library quality as well as subsequent template preparations and sequencing reaction steps were performed following the same protocol used for FFPE samples and WBC. All NGS reactions were run with a mean depth of 1500X ranging of coverage for each amplicon per sample. Sequence results were analyzed by the Torrent Suite Software 5.0.5 and all reads were aligned to the human reference hg19 Genome. The variant calling was performed by the Torrent Variant Caller plugin version 5.0.4.0. Integrative Genomics Viewer (IGV) browser (Broad Institute, Cambridge, Massachusetts, USA) was finally used for interpretation and verification of all sequence variants.

For each patient, the calls obtained from genomic DNA and FFPE samples were compared to those obtained from each of the analyzed CTC sub-populations and, in order to obtain an acceptable quality standard, the variants with a sequencing depth of at least 600X coverage and an allelic frequency of at least 5% were considered, as reported^34,55^. Once the gene variants were detected in the majority of the abovementioned compartments, but not in all of them, BAM files were used to verify their absence in the corresponding loci.

Each variant was investigated in its potential pathogenic role consulting WEB databases as HGMD (http://www.hgmd.cf.ac.uk/ac/index.php), COSMIC (http://cancer.sanger.ac.uk/cosmic), dbSNP (https://www.ncbi.nlm.nih.gov/snp/) and Exome Aggregation Consortium (ExAC) (http://exac.broadinstitute.org/), and the prediction algorithms SIFT, Polyphen and FATHMM.

### Statistical analysis

Analysis of variance (ANOVA) was assessed using Student’s t-test, as well as χ2 or Fisher tests, as appropriate. A p-value ≤ 0.05 was considered significant and analyses were performed using GraphPad Prism 5 software (GraphPad Software, La Jolla, CA, USA).

## Supplementary information

Supplementary Dataset 1

## Data Availability

The datasets generated during and/or analysed during the current study are available from the corresponding author on reasonable request.
