# Supplementary material for: Dissection of major cancer gene variants in subsets of circulating tumor cells in advanced breast cancer
Source: Sci Rep. 2019 Nov 21;9:17276. doi: 10.1038/s41598-019-53660-x (PMC6872745; doi:10.1038/s41598-019-53660-x)
Supplement: Supplementary file 1 — Supplementary Dataset 1 [file 41598_2019_53660_MOESM1_ESM.pdf]

Dissection of major cancer gene variants in subsets of circulating tumor cells in advanced breast cancer

Stella D'Oronzo[1,2§], Domenica Lovero[1§], Raffaele Palmirotta[1],Luigia Stefania Stucci[1], Marco Tucci[1], Claudia Felici [1], Eliano Cascardi[3], Carmela Giardina[3], Paola Cafforio[1],and Franco Silvestris[1*]

[1]Department of Biomedical Sciences and Human Oncology – Section of Internal Medicine and Clinical Oncology
[2]I.R.C.C.S-Giovanni Paolo II Cancer Institute, 70124 Bari, Italy
[3]Department of Emergency and Organs Transplant, Division of Pathology, University of Bari Aldo Moro, P.zza G. Cesare, 11 - 70124 Bari, Italy.

[§]Both authors equally contributed to this work.

*Correspondence**: Franco Silvestris** – francesco.silvestris@uniba.it

Other e-mail addresses:Stella D'Oronzo – stella.doronzo@uniba.it
Domenica Lovero – domenica.lovero@uniba.it
Raffaele Palmirotta – raffaelepalmirotta@gmail.com
Luigia Stefania Stucci - stuccistefania@gmail.com
Marco Tucci - marco.tucci@uniba.it
Claudia Felici – claudiafelici85@live.it
Eliano Cascardi – eliano20@hotmail.it
Carmela Giardina – carmela.giardina@uniba.it
Paola Cafforio – paola.cafforio@uniba.it

Supplementary tables. Sequence variants in major driver genes identified in FFPE primary tumor samples and CTC subsets.

Abbreviations: M+/E-: CTCs expressing only mesenchymal markers; M-/E-: CTCs which do not express both mesenchymal and epithelial markers; M+/E+: CTCs expressing both mesenchymal and epithelial markers; M-/E+: CTCs expressing only epithelial markers; ND: not detectable.

The sequence variants written in red represent pathogenic mutations, while those in orange are of a uncertain clinical significance. The red boxes indicate the presence of the sequence variant, the green boxes indicate the wild type status. For each sequence variant the allele frequency (%) is also reported.

pt. #123							
GENE	Nucleotide variant	Amino acid mutation	COSMIC/dbSNP ID number	gDNA	FFPE	CTC M+/E+	CTC M+/E-
APC	c.4479G>A	p.T1493T	COSM3760869	51.13	51.4	44.10	55.7
APC	**c.4533C>T**	**p.Leu1511**	**rs150089434**			8.00	5.80
AKT1	c.480T>C	p.T160T	-				7.45
AKT1	c.543C>A	p.L181L	-				7.86
AKT1	c.474G>A	p.K159K	-				8.05
AKT1	c.486C>G	p.G162G	-				8.29
ATM	c.1023C>T	p.V341V	rs35728619		24.8		11.3
ATM	**c.7328G>A**	**p.R2443Q**	**COSM20404**		25.5		12.3
CDH1	**c.1043C>T**	**p.A348V**	**-**				5.45
CSF1R	c.*1841TG>GA	p.?	rs386693509	28.00	27.5	38.93	39.7
EGFR	c.2361G>A	p.Q787Q	COSM1451600	99.45	99.6	69.47	99.6
ERBB2	c.2638G>A	p.D880N	COSM7002319				5.32
ERBB4	c.421+58A>G	p.?	rs839541			32.72	
ERBB4	c.729C>T	p.D243D	rs755321591	52.48	44.7	29.09	46.7
FBXW7	c.1737G>A	p.G580G	-				6.00
FBXW7	c.1776T>C	p.N593N	-				6.01
FBXW7	c.1716T>C	p.N572N	-				6.05
FGFR2	**c.1654G>A**	**p.G552R**	**-**				5.85
FGFR3	c.1953G>A	p.T651T	rs7688609	99.58	99.6	99,81	99.7
FLT3	c.1310-3T>C	p.?	rs2491231	48.08	50.3	60.53	55.8
GNA11	c.606-10G>A	p.?	-				5.90
HRAS	c.81T>C	p.H27H	COSM249860	96.22	98.5	60.16	99.7
IDH2	c.483C>T	p.V161V	-				5.75
IDH2	c.477C>T	p.R159R	-				7.05
KDR	c.798+54G>A	p.?	rs7692791	36.89	34.0	60.86	42.7
KDR	c.3849-24C>A	p.?	rs10006115			9.85	
KDR	c.2679T>G	p.G893G	-			5.00	5.33
KIT	c.2538C>T	p.K786K	-			6.50	
MET	**c.2962C>T**	**p.R988C**	**COSM1666978 rs34589476**	46.34	50.1	40.18	43.0
NOTCH1	c.4768T>C	p.F1590L	-				5.04
NOTCH1	c.4702A>C	p.R1568R	-				5.13
NRAS	c.51C>T	p.S17S	-			5.35	
PDGFRA	c.1701A>G	p.P567P	rs1873778	100	99.8	100	99.9
PDGFRA	c.2472C>T	p.V824V	COSM22413	48.27	50.6	32.82	56.6
PIK3CA	c.352+40A>G	p.?	rs3729674			9.35	
PIK3CA	**c.1624G>C**	**p.E542Q**	**COSM17442**		27.4		
PIK3CA	**c.3145G>C**	**p.G1049R**	**COSM12597**		31.6		
PIK3CA	c.3270G>A	p.?	rs183721599			7.06	
PIK3CA	c.3141T>C	p.H1047H	-			6.35	
PIK3CA	c.3081C>T	p.A1027A	-			6.00	
PIK3CA	c.3054C>T	3'UTR	-			6.30	
PIK3CA	c.3282C>T	3'UTR	-			6.50	
PIK3CA	c.3266C>G	3'UTR	-			6.60	
PIK3CA	c.3245C>T	3'UTR	-			6.85	
RET	c.2307G>T	p.L769L	rs1800861	100	99.0	90.23	99.9
RET	c.2712C>G	p.S904S	rs1800836	48.97	44.7	55.81	46.4
SMAD4	c.1335A>G	p. R445R	-			11.00	17.00
SMAD4	c.1389A>G	p.A463A	-			6.96	
SMAD4	c.1410T>C	p.P470P	-			6.81	
SMAD4	c.1332T>C	p.H444H	-			8.94	
SMAD4	c.1059C>T	p.Y353Y	rs863224400				11.3
SMAD4	c.1062G>A	p.V354V	-				11.35
SMAD4	**c.512C>A**	**p.Ser171***	**COSM1151109**				6.16
SMAD4	c.1089T>C	p.C363C	-				11.7
SMAD4	c.1035C>T	p.C345C	-				12.4
SMAD4	c.1002G>A	p.Q334Q	rs773598775				12.5
SMAD4	c.990A>G	p.E330E	-				12.6
SMARCB1	c.1119-41G>A	p.?	COSM1090			30.60	
SMO	c.1623G>A	p.T541T	COSM1085436			25.40	34.5
SMO	c.1566G>A	p.L522L	-			25.78	32.8
SMO	c.1539G>C	p.P513P	-			19.06	33.1
SMO	c.1545T>C	p.L515L	-			26.16	33.9
SMO	c.1590_1591delCGinsTT	p.IA530_531IS	-			25.84	34.2
STK11	**c.976C>A**	**p.P326T**	**rs771632414**	49.77	53.1	39.57	43.9
STK11	c.135C>T	p.L45L	rs1362065365				5.35
STK11	c.174C>G	p.G58G	rs756661578			5.65	5.35
STK11	c.171A>G	p.E57E	rs1060503785			5.70	5.55
STK11	c.177T>G	p.S59S	-			5.00	5.86
STK11	c.72G>A	p.T24T	-			5.30	6.00
STK11	c.141C>A	p.G47G	-			5.71	6.47
STK11	c.84C>G	p.R28R	-			5.00	5.91
STK11	c.114G>C	p.P38P	-			5.41	6.20
TP53	c.215C>G	p.P72R	COSM250061	54.45	49.6	61.36	55.0

pt. #242								
**GENE**	**Nucleotide variant**	**Amino acid mutation**	**COSMIC/dbSNP ID number**	**gDNA**	**CTC M-/E+**	**CTC M+/E+**	**CTC M+/E-**	**CTC M-/E-**
ABL1	c.750G>C	p. G250G	-				15.24	
ABL1	c.774G>A	p.E258E	-				17.64	
ABL1	c.747C>G	p.G249G	-				17.45	
ABL1	c.714A>G	p.E238E	-				17.58	
APC	c.4479G>A	p.T1493T	COSM3760869	50.30	53.94	49.12	50.82	53.24
ATM	**c.1810C>T**	**p.P604S**	**COSM22499**			6,91		
ATM	c.1874A>G	p.N625S	-				8.85	
CDKN2A	c.234C>T	p.H74H	-				13.78	
CSF1R	c.*1841TG>GA	p.?	rs386693509	99.70	99.57	99.41	99.77	100
EGFR	c.2361G>A	p.Q787Q	COSM1451600	99.65	100	100	100	99.45
ERBB4	c.421+58A>G	p.?	rs839541	48.70	54.75	51.35	64.00	49.53
FBXW7	**c1702G>A**	**p.V568M**	**-**				10.60	
FBXW7	c1704G>A	p.V568V	-				10.50	
FBXW7	c.1737G>A	p.G580G	-		8.76		10.60	
FBXW7	c.1776T>C	p.N593N	-		9.00		10.26	
FBXW7	c.1716T>C	p.N572N	-		8.45		10.51	
FGFR3	c.1953G>A	p.T651T	rs7688609	99.86	99.87	99.73	99.70	98.97
FLT3	c.1310-3T>C	p.?	rs2491231	52.55	56.68	57.85	56.90	51.30
HRAS	c.81T>C	p.H27H	COSM249860	50.46	48.79	45,61	50.89	42.46
KDR	c.1416A>T	p.Q47H	COSM149673	14.27			11.70	
KDR	c.2615-36A>CA	p.?	rs3214870	41.91	54.70	39.74	47.30	51.71
KIT	**c.1621A>C**	**p.M541L**	**COSM28026**	51.05	56.51	46,23	38.7	47.71
NRAS	**c.181C>A**	**p.Q61K**	**COSM580**		9.15			
PDGFRA	**c.1667A>G**	**p.E556G**	**-**				8.66	
PDGFRA	c.2017A>C	p.I673L	-				8.82	
PDGFRA	c.1701A>G	p.P567P	rs1873778	99.90	99.90	100	100	99.80
PIK3CA	**c.323G>A**	**p.R108H**	**COSM27497**				5.69	
PIK3CA	c.352+40A>G	p.?	rs3729674	49.85	51.93	54.80	34.34	48.78
PTEN	c.324T>C	p.L108L	-				9.43	
RET	c.1908G>A	p.T382T	rs886038663				9.98	
RET	c.2307G>T	p.L769L	rs1800861	48.97	41,55	51.89	67.86	52.03
SMAD4	c.727G>A	p.G243R	**-**				10.75	
SMAD4	**c.1216G>A**	**p.A406T**	**COSM10103**				11.76	
SMAD4	c.1335A>G	p. R445R	-		14.00	5.23	10.00	8.00
SMAD4	c.1059C>T	p.Y353Y	rs863224400				13.06	
SMAD4	c.1062G>A	p.V354V	-				12.86	
SMAD4	c.1089T>C	p.C363C	-				13.00	
SMAD4	c.1035C>T	p.C345C	-				13.07	
SMAD4	c.1002G>A	p.Q334Q	rs773598775				13.12	
SMAD4	c.990A>G	p.E330E	-				13.51	
SMO	**c.1214T>C**	**p.L405P**	**-**		6.72			
SMO	c.1623G>A	p.T541T	COSM1085436		6.38		12.48	
SMO	c.1566G>A	p.L522L	-		6.58		12.21	
SMO	c.1539G>C	p.P513P	-		6.62		12.35	
SMO	c.1545T>C	p.L515L	-		6.74		12.32	
SMO	c.1590_1591del CGinsTT	p.IA530_531IS	-		7.00		12.05	
STK11	c.465-51T>C	p.?	rs2075606				18.51	
STK11	**c.536C>T**	**p.P179L**	**COSM51520**				10.87	
STK11	c.174C>G	p.G58G	rs756661578		9.31			
STK11	c.171A>G	p.E57E	rs1060503785		9.35			
STK11	c.177T>G	p.S59S	-		9.37			
STK11	c.72G>A	p.T24T	-		9.35			
STK11	c.141C>A	p.G47G	-		9.46			
STK11	c.84C>G	p.R28R	-		9.40			
STK11	c.114G>C	p.P38P	-		10.21			
TP53	**c.388C>A**	**p.L130I**	**IARCTP53**		5.00		5.96	
TP53	c.215C>G	p.P72R	COSM250061	98.40	97.51	97.10	97.12	96.38
TP53	c.1043T>C	p.L348S	-				5.43	
VHL	c.378T>C	p.D126D	-				6.10	

pt. #253							
**GENE**	**Nucleotide variant**	**Amino acid mutation**	**COSMIC/dbSNP ID number**	**gDNA**	**CTC M-/E+**	**CTC M+/E+**	**CTC M-/E-**
APC	c.4479G>A	p.T1493T	COSM3760869	*100*	*98.09*	*nc*	*97.09*
APC	c.2601A>G	p.T867T	-			*9.57*	
ATM	c.9159A>G	p.K3053K	rs1060504315			*20.42*	
CDH1	**c.1027C>A**	p.L343M	-			*19.64*	
CSF1R	c.*1841TG>GA	p.?	rs386693509	*99.51*	*99.78*	*21.94*	*99.24*
EGFR	c.2361G>A	p.Q787Q	COSM1451600	*51,94*	*51.70*	*nc*	*66.73*
ERBB4	**c.1080C>T**	p.T360T	rs183921316			*6.15*	
FBXW7	**c.1451G>T**	**p.R484M**	**COSM34016**			*39.00*	
FGFR1	c.518A>G	p.Glu173Gly	-			*27.20*	
FGFR3	c.1953G>A	p.T651T	rs7688609	*100*	*99.59*	*99,38*	*99.60*
FGFR3	**c.1150T>C**	**p.F384L**	**COSM724 rs17881656**	*45.35*	*44.89*	*nc*	*53.27*
FLT3	c.1310-3T>C	p.?	rs2491231	*53.02*	*36.50*	*99.53*	*27.56*
HNF1A	c.635A>G	p.Q212R	-			*52.45*	
HRAS	c.81T>C	p.H27H	COSM249860			*52.68*	
JAK3	c.2156T>C	p.F719S	-			*17.86*	
KDR	c.798+54G>A	p.?	rs7692791	*48.00*	*40.91*	*nc*	*37.64*
KDR	c.1416A>T	p.Q47H	COSM149673	*99.82*	*99.75*		*99.66*
KDR	c.2615-36A>CA	p.?	rs3214870	*100*	*99.80*		*100*
KIT	c.1936T>C	p.Y646H	COSM3734987			*5.34*	
PDGFRA	c.1701A>G	p.P567P	rs1873778	*100*	*100*	*nc*	*100*
PIK3CA	**c.1633G>A**	**p.E545K**	**COSM763**		*48.63*		*53.55*
PIK3CA	**c.3140A>T**	**p.H1047L**	**COSM776**		*59.35*		*68.78*
PIK3CA	c.1348C>T	p.H450Y	-			*18.75*	
PIK3CA	c.3076C>T	p.(=)	-			*6.06*	
RET	c.2307G>T	p.L769L	rs1800861			*99.58*	
SMARCB1	c.1119-41G>A	p.?	COSM1090	*49.38*	*32.62*		*25.79*
SMO	c.623A>G	p.E208G	-			*5.88*	
SMO	**c.646C>T**	**p.Q216Ter**	**COSM6906070**			*11.57*	
STK11	c.465-51T>C	p.?	rs2075606	*49.67*	*37.45*	*nc*	*33.20*
TP53	c.215C>G	p.P72R	COSM250061	*50.77*	*52.39*	*nc*	*38.33*
TP53	**c.742C>T**	**p.R248TW**	**COSM10656**		*20.19*	*nc*	*38.45*

pt. #274							
**GENE**	**Nucleotide variant**	**Amino acid mutation**	**COSMIC/dbSNP ID number**	**gDNA**	**FFPE**	**CTC M+/E+**	**CTC M+/E-**
CSF1R	c.*1841TG>GA	p.?	rs386693509	*26.5*	*41.8*	*33.93*	*32.2*
EGFR	c.2361G>A	p.Q787Q	COSM1451600	*51.8*	*51.4*	*52.76*	*53.4*
ERBB4	c.421+58A>G	p.?	rs839541	*48.5*	*55.0*	*51.43*	*46.9*
FGFR3	c.1953G>A	p.T651T	rs7688609	*99.5*	*99.5*	*99.70*	*99.6*
HRAS	c.81T>C	p.H27H	COSM249860	*52.2*	*53.6*	*48.09*	*48.9*
IDH1	c.315C>T	p.G105G	rs11554137	*50.4*	*44.9*	*43.17*	*47.0*
KDR	c.798+54G>A	p.?	rs7692791	*99.9*	*96.9*	*100*	*99.6*
KDR	c.4035T>C	p.P1345P	-				*16.4*
PDGFRA	c.1701A>G	p.P567P	rs1873778	*100*	*99.9*	*100*	*99.9*
RET	c.2307G>T	p.L769L	rs1800861	*100*	*99.5*	*99.95*	*99.9*
RET	c.2712C>G	p.S904S	rs1800836	*46.9*	*46.7*	*49.65*	*43.9*
SMAD4	c.955+58C>T	p.?	rs948588	*51,5*	*48.4*	*40.70*	*48.8*
TP53	c.215C>G	p.P72R	COSM250061	*51.3*	*63.4*	*59.16*	*50.7*
TP53	**c.833C>T**	**p.P278L**	**COSM10863**		*13.8*		

pt. #279								
**GENE**	**Nucleotide variant**	**Amino acid mutation**	**COSMIC/dbSNP ID number**	**gDNA**	**FFPE**	**CTC M+/E+**	**CTC M+/E-**	**CTC M-/E-**
APC	c.4479G>A	p.T1493T	COSM3760869				*10.07*	*11.27*
ATM	**c.1810C>T**	**p.P604S**	**COSM22499**				*7.31*	*11.1*
CSF1R	c.*1841TG>GA	p.?	rs386693509	*100*	*100*	*99.8*	*82.94*	*75.3*
EGFR	c.2361G>A	p.Q787Q	COSM1451600	*99.40*	*99.2*	*99.5*	*98.02*	*100*
EGFR	**c.2257C>T**	**p.P753S**	**COSM6268**					*5.14*
ERBB4	c.421+51C>T	p.?	-					*7.86*
ERBB4	c.421+58A>G	p.?	rs839541	*99.31*	*99.7*	*99.9*	*81.90*	*90.1*
FGFR3	c.1953G>A	p.T651T	rs7688609	*99.68*	*99.5*	*99.7*	*99.75*	*99.7*
FGFR3	c.1959+22G>A	p.?	rs3135898	*47.50*	*46.9*	*40.3*	*32.29*	*40.2*
FGFR3	**c.2408G>A**	**p.G803D**	**COSM7208599**			*6.95*		
FGFR3	**c.1150T>C**	**p.F384L**	**COSM724**				*6.91*	
FLT3	c.1310-3T>C	p.?	rs2491231	*51.05*	*50.9*	*44.1*	*59.03*	*60.4*
HNF1A	**c.620G>A**	**p.G207D**	**COSM24931**			*5.05*		
HRAS	c.81T>C	p.H27H	COSM249860				*6.14*	
IDH1	c.315C>T	p.G105G	rs11554137				*5.50*	
KDR	c.798+54G>A	p.?	rs7692791				*8.60*	*5.00*
KIT	**c.1621A>C**	**p.M541L**	**COSM28026**	*50.90*	*48.5*	*30.9*	*32.52*	*39.5*
MPL	c.1565+27G>A	p.?	-			*6.15*		
PDGFRA	c.1701A>G	p.P567P	rs1873778	*100*	*99.4*	*100*	*99.94*	*100*
PDGFRA	c.2472C>T	p.V824V	COSM22413				*5.05*	*9.00*
PIK3CA	c.1173A>G	p.I391M	rs2230461	*54.45*	*60.5*	*49.5*	*54.09*	*61.4*
PIK3CA	c.352+40A>G	p.?	rs3729674	*52.23*	*48.5*	*42.6*	*45.49*	*41.6*
PIK3CA	**c.3140A>T**	**p.H1047L**	**COSM776**		*29.5*			
RET	c.2307G>T	p.L769L	rs1800861	*99.80*	*99.9*	*100*	*93.97*	*96.2*
RET	c.2712C>G	p.S904S	rs1800836	*47.62*	*46.5*	*56.8*	*47.29*	*50.1*
SMAD4	c.1059C>T	p.Y353Y	rs863224400					*5.10*
SMARCB1	c.1119-41G>A	p.?	COSM1090	*48.10*	*72.7*	*63.3*	*39.30*	*46.5*
SMO	c.1623G>A	p.T541T	COSM1085436				*5.26*	
SMO	c.1566G>A	p.L522L	-				*5.41*	
SMO	c.1539G>C	p.P513P	-				*5.40*	
SMO	c.1545T>C	p.L515L	-				*5.40*	
STK11	c.465-51T>C	p.?	rs2075606				*14.79*	*10.7*
STK11	c.80A>G	p.H27R	-				*5.25*	
TP53	c.215C>G	p.P72R	COSM250061	*97.85*	*96.4*	*98.4*	*98.65*	*86.4*

pt. #335						
**GENE**	**Nucleotide variant**	**Amino acid mutation**	**COSMIC/dbSNP ID number**	**gDNA**	**FFPE**	**CTC M+/E-**
ABL1	c.750G>C	p. G250G	-			*7.80*
ABL1	c.774G>A	p.E258E	-			*10.0*
ABL1	c.747C>G	p.G249G	-			*9.00*
ABL1	c.714A>G	p.E238E	-			*9.70*
APC	c.4479G>A	p.T1493T	COSM3760869	*51.8*	*51.9*	*57.3*
APC	**c.4348C>T**	**p.R1450***	**COSM13127**			*5.25*
AKT1	c.48G>T	p.G16G	-		*43.8*	
ATM	**c.7328G>A**	**p.R2443Q**	**COSM20404**			*12.20*
CSF1R	c.*1841TG>GA	p.?	rs386693509	*99.8*	*97.0*	*64.0*
EGFR	c.2361G>A	p.Q787Q	COSM1451600		*6.17*	
ERBB4	c.421+58A>G	p.?	rs839541			*10.0*
FGFR3	c.1953G>A	p.T651T	rs7688609	*99.8*	*99.4*	*96.3*
FLT3	c.1310-3T>C	p.?	rs2491231	*100*	*96.7*	*64.5*
HRAS	c.81T>C	p.H27H	COSM249860	*98.9*	*95.8*	*97.5*
KDR	c.798+54G>A	p.?	rs7692791	*47*	*38*	*49.8*
KDR	c.4035T>C	p.P1345P	-			*5.36*
KDR	c.4008C>T	p.T1336T	COSM35855	*52.4*	*48.0*	*26.5*
KDR	c.4022A>G	p.Q1341R	-			*5.01*
KDR	**c.2946C>T**	**p.S982S**	**COSM4992129**			*5.75*
MET	**c.2962C>T**	**p.R988C**	**COSM1666978**			*9.0*
PDGFRA	c.1701A>G	p.P567P	rs1873778	*100*	*99.7*	*99.8*
PDGFRA	c.2472C>T	p.V824V	COSM22413			*26.4*
PTEN	**c.959T>A**	**p.L320***	**COSM35671**		*62.7*	*6.06*
RET	c.2307G>T	p.L769L	rs1800861	*46,25*	*35.1*	*68.6*
RET	c.2712C>G	p.S904S	rs1800836			*6.05*
SMO	c.1623G>A	p.T541T	COSM1085436			*6.25*
SMO	c.1539G>C	p.P513P	-			*6.05*
SMO	c.1545T>C	p.L515L	-			*6.30*
STK11	c.465-51T>C	p.?	rs2075606		*11.7*	
STK11	**c.976C>A**	**p.P326T**	**rs771632414**			*10.10*
STK11	c.598-14C>T	p.?	rs769378511			*5.45*
TP53	c.215C>G	p.P72R	COSM250061	*51,3*	*44.1*	*62.1*
TP53	**c.4G>A**	**p.Glu2Lys**	rs769884991			*5.95*
VHL	c.321C>A	p.R107R	-			*6.04*

pt. #337							
GENE	Nucleotide variant	Amino acid mutation	COSMIC/dbSNP ID number	gDNA	FFPE	CTC M+/E+	CTC M+/E-
ALK	c.3836+1G>T	Splice site	-			5.71	
APC	c.4479G>A	p.T1493T	COSM3760869	99.82	99.8	100	100
APC	c.4114C>A	p.P1372T	-			5.38	
AKT1	c.528C>A	p.Tyr176*	-				6.19
ATM	c.8177C>T	p.Ala2726Val	-				6.80
ATM	c.3878A>G	p.N1293S	COSM5490564				6.95
ATM	c.2578G>T	p.Asp860Tyr	-				8.32
ATM	c.3947C>T	p.Thr1316Ile	-				9.05
BRAF	c.1379G>T	p.Gly460Val	rs121913351				6.29
CDH1	c.1097C>A	p.T366N	-			12.02	
CSF1R	c.*1841TG>GA	p.?	rs386693509	28.43	51.5	29.54	45.2
EGFR	c.2361G>A	p.Q787Q	COSM1451600	53.30	48.1	74.02	49.00
EGFR	c.2469+32G>T	p.?	-				11.8
ERBB2	c.2527C>T	p.His843Tyr	-				8.74
ERBB2	c.2551G>T	p.V851L	-			5.04	
ERBB4	c.421+58A>G	p.?	rs839541			6.98	
ERBB4	c.1084A>G	p.I362V	-				6.32
ERBB4	c.1015A>G	p.T339A	-				7.04
ERBB4	c.915G>A	p.V305V	rs781470325				7.68
FGFR1	c.497_498insTGA	p.D166dup	rs776622310	30.26	42.5	29.50	41.1
FGFR2	c.1130A>G	p.E377G	-				6.97
FGFR2	c.939+41C>A	p.?	-				11.0
FGFR3	c.1953G>A	p.T651T	rs7688609	99.51	99.6	100	99.9
FLT3	c.1310-3T>C	p.?	rs2491231	51.17	51.7	76.10	47.5
FLT3	c.2001G>T	p.Q667H	-			10.95	
FLT3	c.2537G>T	p.G846V	-			8.30	
HNF1A	c.771C>A	p.N257K	-				5.91
HRAS	c.60C>T	p.T20T	ExAC			8.93	
IDH1	c.354C>T	p.P118P	ExAC			9.10	
KDR	c.798+54G>A	p.?	rs7692791	38.37	40.7	27.91	36.6
KDR	c.1416A>T	p.Q47H	COSM149673	51.34	39.1	33.05	72.8
KDR	c.3849-24C>A	p.?	rs10006115	54.95	50.9	51.68	36.7
KDR	c.3454A>G	p.T1152A	-				6.62
KDR	c.2615-36A>CA	p.?	rs3214870	56.6	49.7	33.54	38.5
KDR	c.2654A>G	p.E885G	-			8.64	
KIT	c.1904A>G	p.E635G	COSM144512				5.74
MET	c.3394+3A>G	p.?	COSM5731184				8.22
NOTCH1	c.5026G>A	p.V1676I	COSM327182				10.6
NOTCH1	c.5019-4C>A	p.?	-				6.95
NOTCH1	c.7362C>T	p.H2454H	-			7.33	
NRAS	c.394G>T	p.E132Ter	-			5.17	
PDGFRA	c.1701A>G	p.P567P	rs1873778	100	100	100	100
PDGFRA	c.2472C>T	p.V824V	COSM22413	47.9	55.1	41.78	70.0
PDGFRA	c.1955G>T	p.G652V	COSM3946437			5.47	
PIK3CA	c.352+40A>G	p.?	rs3729674	51.88	46.5	48.60	62.7
PIK3CA	c.352+1G>A	Splice site	-			7.01	
PIK3CA	c.3196G>A	p.A1066T	COSM6941468			18.0	64.0
PIK3CA	c.1238A>G	p.K413R	-				8.04
PTEN	c.63C>A	p.F21L	-				7.24
PTEN	c.209+45A>G	p.?	-			5.17	
PTEN	c.1008C>T	p.Y336Y	-			7.76	
PTPN11	c.1578G>A	p.Q526Q	-			5.62	
PTPN11	c.1478C>A	p.T493N	-			5.90	
RB1	c.1389+37C>T	p.?	rs373682367			5.01	
RET	c.2307G>T	p.L769L	rs1800861	100	100	100	99.9
RET	c.2712C>G	p.S904S	rs1800836	51.67	43.5	46.66	45.5
RET	c.2681G>T	p.G894V	-				5.97
SMAD4	c.949C>A	p.H317AN	-			7.22	
SMAD4	c.955+58C>T	p.?	rs948588	50.95	58.4	24.21	66.8
SMAD4	c.573G>T	p.S191S	COSM6475980				5.60
SMO	c.1545T>C	p.L515L	-			5.66	
SMO	c.1590_1591delCGinsTT	p.IA530_531IS	-				
SMO	c.1592C>T	p.A531V	-			5.35	
SMO	c.677A>G	p.E226G	-				5.50
SMO	c.614G>A	p.S205N	-				6.40
STK11	c.465-51T>C	p.?	rs2075606	47.93	43.9	65.61	60.5
TP53	c.215C>G	p.P72R	COSM250061	97.99	97.2	98.78	99.1

pt. #371							
GENE	Nucleotide variant	Amino acid mutation	COSMIC/dbSNP ID number	gDNA	FFPE	CTC M+/E-	CTC M-/E-
APC	c.4479G>A	p.T1493T	COSM3760869	*99.78*	*97.7*	*88.0*	*87.3*
APC	c.4548A>T	p.I1516I	-			*6.76*	
APC	c.4524T>C	p.A1508A	rs767025305			*6.95*	
APC	c.4512C>T	p.S1504S	-			*7.40*	
APC	c.4476C>T	p.A1492A	-			*7.84*	
ATM	**c.1810C>T**	**p.P604S**	**COSM22499**			*6.66*	
CSF1R	c.*1841TG>GA	p.?	rs386693509	*34.48*	*23.50*	*24.4*	*31.6*
EGFR	c.2361G>A	p.Q787Q	COSM1451600	*48.80*	*51.6*	*59.7*	*48.1*
ERBB2	c.2638G>A	p.D880N	COSM7002319				*5.02*
ERBB2	c.2642G>A	p.G881E	-				*5.19*
ERBB2	c.2644G>T	p.G882C	-				*5.41*
FGFR3	c.1953G>A	p.T651T	rs7688609	*100*	*99.6*	*99.6*	*99.5*
FLT3	c.1310-3T>C	p.?	rs2491231	*100*	*100*	*100*	*100*
HRAS	c.81T>C	p.H27H	COSM249860	*48.50*	*58.0*	*60.6*	*49.3*
KDR	c.798+54G>A	p.?	rs7692791	*53.00*	*43.00*	*45.4*	*49.00*
KIT	c.103T>C	p.S35P	-				*5.20*
KIT	**c.1638A>G**	**p.K546K**	**COSM21983**	*44.84*	*50.8*	*50.7*	*53.6*
MET	c.3798G>A	p.K1266K	**-**			*6.46*	
PIK3CA	c.1173A>G	p.I391M	rs2230461			*8.50*	
PIK3CA	c.352+40A>G	p.?	rs3729674	*45.36*	*50.3*	*43.1*	*39.8*
PIK3CA	**c.1633G>A**	**p.E545K**	**COSM763**		*20.7*		
PTEN	**c.991G>A**	**p.D331N**	**COSM6327339**			*7.90*	
PTEN	**c.635-1G>A**	**p.?**	**COSM28920**			*6.75*	
PTPN11	c.193C>T	p.L65L	-			*5.45*	
PTPN11	**c.169C>T**	**p.Q57***	**-**			*6.00*	
RET	c.2307G>T	p.L769L	rs1800861	*44.58*	*41.6*	*53.2*	*53.3*
SMAD4	**c.767A>T**	**p.Q256L**	**COSM5546625**				*11.2*
SMAD4	c.1335A>G	p. R445R	-			*10.0*	*10.0*
SMO	c.1623G>A	p.T541T	COSM1085436			*5.15*	
SMO	c.1566G>A	p.L522L	-			*5.16*	
SMO	c.1539G>C	p.P513P	-			*5.65*	
SMO	c.1545T>C	p.L515L	-			*5.70*	
STK11	c.465-51T>C	p.?	rs2075606	*56.80*	*46.0*	*41.2*	*61.7*
TP53	**c.388C>A**	**p.L130I**	**IARCTP53**				*9.70*
TP53	c.215C>G	p.P72R	COSM250061	*97.13*	*100*	*98.2*	*95.7*

pt. #372							
GENE	Nucleotide variant	Amino acid mutation	COSMIC/dbSNP ID number	gDNA	CTC M-/E+	CTC M+/E+	CTC M+/E-
ABL1	c.720G>A	p.T240T	-		9.66		
APC	c.4479G>A	p.T1493T	COSM3760869	49.77	71.74	47.17	52.03
APC	c.2613A>G	p.G871G	-				9.78
APC	c.2652A>G	p.A884A	-				9.06
APC	c.3362T>C	p.I1121T	-		26.48		
AKT1	c.489G>A	p.k163K	rs576374556		9.05		
ATM	**c.1810C>T**	**p.P604S**	**COSM22499**		65.97	14.34	
ATM	c.3927A>G	p.A1309A	rs1555093665				6.15
ATM	**c.5056A>G**	**p.I1686V**	**rs145453814**				5.05
ATM	c.5381T>C	p.L1794P	-				9.80
CDH1	**c.254T>C**	**p.V85A**	**rs878854688**				10.95
CDH1	c.1078A>G	p.T360A	-				5.94
CDH1	c.237A>G	p.T79T	-				7.00
CDKN2A	c.310C>T	p.L104V	-		32.16		
CSF1R	c.*1841TG>GA	p.?	rs386693509	99.89	100	37.36	46.42
EGFR	c.2361G>A	p.Q787Q	COSM1451600	99.60	100	99.39	97.08
EGFR	**c.2300C>T**	**p.A767V**	**COSM26445**		11.27		
EGFR	c.2469+57C>T	p.?	-				5.35
EGFR	c.2469+27G>A	p.?	rs368440941				5.85
EGFR	c.2459A>G	p.Q820R	COSM85898				5.95
EGFR	c.2436G>A	p.I821I	-		16.05		
EGFR	c.875T>C	p.V292A	ExAC		6.67		
ERBB2	c.2307+46A>G	p.?	rs4252645		5.30		
ERBB2	c.2307+66T>A	p.?	-		5.45		
ERBB4	c.421+58A>G	p.?	rs839541		28.25	47.47	47.85
FBXW7	c.1319A>G	p.Asp440Gly	-		9.89		
FBXW7	c.861+17A>G	p.?	-		13.35		
FBXW7	**c.1442C>T**	**p.A481V**	**COSM4122764**				7.10
FGFR3	c.1953G>A	p.T651T	rs7688609	99.80	100	99,58	99.61
FGFR3	c.1959+22G>A	p.?	rs3135898	55.02		25.04	32.14
FGFR3	**c.1150T>C**	**p.F384L**	**COSM724**		41.91	8.77	
FLT3	c.1310-3T>C	p.?	rs2491231	99.75	*nc*	93.75	94.15
FLT3	c.2541+4A>G	p.?	-		24.06		
FLT3	c.2541+11T>C	p.?	-		6.58		
GNAQ	**c.735+1G>T**	**Splice site**	**-**				5.75
HNF1A	c.612C>T	p.F204F	-				5.85
HNF1A	c.638T>C	p.I213T	-				6.31
HRAS	c.81T>C	p.H27H	COSM249860	51.49		26.70	27.28
JAK3	**c.2126G>A**	**p.Trp709Ter**	**-**		20.90		
KDR	c.798+54G>A	p.?	rs7692791		18.09	23.09	9.45
KDR	c.799C>T	p.His267Tyr	?		5.95		
KDR	c.1416A>T	p.Q47H	COSM149673	49.32		40.06	50.97
KDR	c.3849-24C>A	p.?	rs10006115				
KDR	c.2615-36A>CA	p.?	rs3214870	43.08	24.60	48.35	43.31
KDR	c.2881C>A	p.R961R	-				5.00
KIT	**c.1621A>C**	**p.M541L**	**COSM28026**	53.18		12.74	22.78
KIT	c.2019T>C	p.C679C	-		5.50		
KIT	c.2555T>C	p.V852A	ExAC				5.10
KRAS	**c.103A>G**	**p.T35A**	**COSM1360858**		8.30		
KRAS	c.113A>G	p.Asp38Gly	-				5.10
KRAS	**c.153T>C**	**p.C51C**	**COSM5009217**				8.16
MET	c.3770A>G	p.N1257S	-				10.24
MET	c.1115A>G	p.D372G	-				16.53
MET	**c.1124A>G**	**p.N375S**	**COSM5020653**				11.24
MET	c.3794T>C	p.V1265A	-				9.82
MLH1	**c.1184C>T**	**p.A395V**	**rs1064794142**		65.51		
MLH1	c.1156A>G	p.T386A	-				5.25
NOTCH1	c.5019-53G>T	p.?	-		5.25		
NOTCH1	c.7407A>G	p.P2479P	-		6.18		
PDGFRA	c.1701A>G	p.P567P	rs1873778	99.90	99.95	100	100
PDGFRA	c.2472C>T	p.V824V	COSM22413		26.2	34.39	29.45
PDGFRA	c.1990T>C	p.C664R	-				5.50
PIK3CA	c.1173A>G	p.I391M	rs2230461	54.55	24.40		
PIK3CA	c.266T>C	p.L89P	-		7.25		
PIK3CA	c.352+40A>G	p.?	rs3729674	49.60			
PIK3CA	c.240A>G	p.E80E	-		8.85		
PIK3CA	**c.3140A>G**	**p.H1047R**	**COSM775**			9.41	7.30
PIK3CA	c.2752A>G	p.N918D	-				5.05
PTEN	c.995A>G	p.L332R	-		10.73		
PTEN	c.60A>G	p.G20G	COSM4016447		5.44		
PTEN	c.966A>G	p.K322K	rs786201392		23.03		
RB1	c.1724A>G	p.Q575R	-		6.20		
RB1	**c.1975T>C**	**p.Y659H**	**COSM327254**		6.30		
RB1	c.2271C>T	p.N757N	-		6.73		
RB1	c.2253T>C	p.S751S	rs373070661		6.74		
RB1	c.2259A>C	p.I753I	-		6.75		
RB1	c.2232C>T	p.I744I	-		6.79		
RB1	c.2234A>G	p.K745R	-		7.75		
RB1	c.1050-30A>G	p.?	-				13.91
RB1	c.2293A>G	p.K765E	-				6.81
RET	c.2307G>T	p.L769L	rs1800861	49.50	100	73.37	78.06
SMAD4	c.1332T>C	p.H444H	-				5.72
SMAD4	c.1176A>G	p.K392K	-		16.53		
SMAD4	c.525A>G	p.E175E	rs368528856				5.66
SMARCB1	c.172G>A	p.E58K	-		15.28		
SMO	**c.1870A>T**	**p.Lys624Ter**	**-**		8.52		
SMO	c.1625T>C	p.L542P	-				8.18
SMO	**c.1180T>C**	**p.T394H**	**COSM3878023**				14.17
SMO	c.929A>G	p.E310G	-				9.66
STK11	c.465-51T>C	p.?	rs2075606		75.48	50.34	19.92
STK11	c.825G>A	p.P275P	rs202011521		11.67		
STK11	c.524A>G	p.K175R	-		9.55		
TP53	**c.388C>A**	**p.L130I**	**IARCTP53**		5.35		5.27
TP53	**c.742C>T**	**p.R248TW**	**COSM10656**			11.25	
TP53	c.311A>G	p.Q104R	-		8.50		
TP53	c.376-24T>C	p.?	-		5.50		
TP53	c.376-20T>C	p.?	rs1483487754				5.65
TP53	**c.667C>T**	**p.P223S**	**COSM44816**				6.42
VHL	c.303G>C	p.L101L	ExAC		10.45		

pt. #382							
**GENE**	**Nucleotide variant**	**Amino acid mutation**	**COSMIC/dbSNP ID number**	**gDNA**	**CTC M+/E+**	**CTC M+/E-**	**CTC M-/E-**
APC	c.4479G>A	p.T1493T	COSM3760869	*99.85*	*100*	*89.97*	*99.85*
CSF1R	c.*1841TG>GA	p.?	rs386693509	*100*	*99.52*	*99.-72*	*99.90*
EGFR	c.2361G>A	p.Q787Q	COSM1451600	*99.70*	*99.63*	*92.89*	*98.98*
ERBB2	c.2270A>G	p.E757G	-			*5.15*	
ERBB2	c.2295A>C	p.K765N	-			*5.17*	
FBXW7	c.1737G>A	p.G580G	-		*6.36*		
FBXW7	c.1776T>C	p.N593N	-		*6.25*		
FBXW7	c.1716T>C	p.N572N	-		*6.45*		
FGFR3	c.1953G>A	p.T651T	rs7688609	*100*	*99.77*	*99.21*	*99.72*
FGFR3	**c.1150T>C**	**p.F384L**	**COSM724**			*5.56*	*7.64*
FLT3	c.1310-3T>C	p.?	rs2491231		*7.20*	*9.95*	
FLT3	c.2510T>A	p.M837K	-			*5.46*	
HRAS	c.81T>C	p.H27H	COSM249860	*47.52*	*49.78*	*46.28*	*38.71*
IDH1	c.315C>T	p.G105G	rs11554137	*49.85*	*52.4*	*41.65*	*44.50*
KDR	c.798+54G>A	p.?	rs7692791	*33.53*	*42.58*	*39.05*	*38.12*
KDR	c.1416A>T	p.Q47H	COSM149673	*49.40*	*54.73*	*40.72*	*50.63*
KDR	c.2615-36A>CA	p.?	rs3214870	*46.13*	*55.61*	*41.79*	*40.00*
KRAS	c.102A>G	p.R102R	-			*6.80*	
NRAS	c.51C>T	p.S17S	-		*7.06*		
PDGFRA	c.1701A>G	p.P567P	rs1873778	*99.90*	*100*	*99.95*	*100*
PIK3CA	c.1173A>G	p.I391M	rs2230461	*58.65*	*50.48*	*38.82*	*46.45*
PIK3CA	c.352+40A>G	p.?	rs3729674	*47.55*	*53.73*	*43.62*	*45.81*
PIK3CA	**c.3196G>A**	**p.A1066T**	**COSM6941468**		*7.0*	*8.66*	
PIK3CA	**c.3140A>G**	**p.H1047R**	**rs121913279**		*5.00*	*5.00*	
RET	c.2307G>T	p.L769L	rs1800861	*99.85*	*99.15*	*89.29*	*99.95*
RET	c.2712C>G	p.S904S	rs1800836	*50.40*	*49.20*	*41.37*	*46.12*
SMAD4	c.955+58C>T	p.?	rs948588			*12.00*	
SMO	c.1623G>A	p.T541T	COSM1085436		*21.10*		
SMO	c.1566G>A	p.L522L	-		*20.91*		
SMO	c.1539G>C	p.P513P	-		*19.30*		
SMO	c.1545T>C	p.L515L	-		*20.85*		
SMO	c.1590_1591del CGinsTT	p.IA530_531IS	-		*21.55*		
STK11	c.465-51T>C	p.?	rs2075606	*99.95*	*82.13*	*86.65*	*84.52*
TP53	c.215C>G	p.P72R	COSM250061	*51.28*	*54.98*	*53.68*	*54.66*
TP53	**c.1009C>T**	**p.R337C**	**COSM11071**		*11.5*		

pt. #392							
**GENE**	**Nucleotide variant**	**Amino acid mutation**	**COSMIC/dbSNP ID number**	**gDNA**	**FFPE**	**CTC M+/E-**	**CTC M-/E-**
ABL1	c.750G>C	p. G250G	-			*21.7*	
ABL1	c.774G>A	p.E258E	-			*23.0*	
ABL1	c.747C>G	p.G249G	-			*23.8*	
ABL1	c.714A>G	p.E238E	-			*24.4*	
APC	c.4479G>A	p.T1493T	COSM3760869	*49.41*	*61.9*	*99.5*	*50.0*
EGFR	c.2361G>A	p.Q787Q	COSM1451600	*99.71*	*99.9*	*nd*	*100*
ERBB4	**c.866G>T**	**p.C289F**	**-**			*22.3*	
ERBB4	c.421+58A>G	p.?	rs839541	*51.06*	*42.5*	*nd*	*51.7*
FGFR3	c.1953G>A	p.T651T	rs7688609	*100*	*99.8*	*99.7*	*100*
FLT3	c.1310-3T>C	p.?	rs2491231	*99.76*	*100*	*nd*	*100*
HRAS	c.81T>C	p.H27H	COSM249860	*51.75*	*50.5*	*nd*	*33.7*
KDR	c.798+54G>A	p.?	rs7692791	*48.00*	*23.7*	*nd*	*34.00*
KIT	c.68-13T>G	p.?	-			*19.5*	
NOTCH1	c.5019-85T>C	p.?	-			*5.04*	
PDGFRA	c.1701A>G	p.P567P	rs1873778	*100*	*100*	*100*	*100*
PIK3CA	c.352+40A>G	p.?	rs3729674			*13.89*	
PIK3CA	**c.1633G>A**	**p.E545K**	**COSM763**			*82.9*	
RET	c.2307G>T	p.L769L	rs1800861	*100*	*100*	*64.0*	*100*
SMAD4	c.1335A>G	p. R445R	-			*100*	
SMAD4	c.1389A>G	p.A463A	-			*100*	
SMAD4	c.1410T>C	p.P470P	-			*100*	
SMAD4	c.594A>G	p.P198P	-			*26.1*	
SMAD4	c.1332T>C	p.H444H	-			*96.7*	
SMAD4	c.1353_1359 delGGCTACTinsAGCCACG	p.AAT451_453AAT	-			*100*	
SMO	c.1623G>A	p.T541T	COSM1085436			*100*	
SMO	c.1566G>A	p.L522L	-			*100*	
SMO	c.1539G>C	p.P513P	-			*95.5*	
SMO	c.1545T>C	p.L515L	-			*99.2*	
SMO	c.1590_1591delCGinsTT	p.IA530_531IS	-			*100*	
STK11	c.465-51T>C	p.?	rs2075606	*52.24*	*53.3*	*nd*	*55*
STK11	c.75C>T	p.F25F	-			*5.2*	
TP53	c.215C>G	p.P72R	COSM250061	*99.40*	*97.8*	*98.0*	*100*

pt. #399							
**GENE**	**Nucleotide variant**	**Amino acid mutation**	**COSMIC/dbSNP ID number**	**gDNA**	**FFPE**	**CTC M+/E-**	**CTC M-/E-**
APC	c.4479G>A	p.T1493T	COSM3760869	*99.8*	*99.8*	*99.8*	*100*
CSF1R	c.*1841TG>GA	p.?	rs386693509	*100*	*98.1*	*99.6*	*99.9*
EGFR	c.2361G>A	p.Q787Q	COSM1451600	*99.6*	*99.7*	*99.5*	*100*
FGFR3	c.1953G>A	p.T651T	rs7688609	*99.7*	*99.7*	*99.8*	*99.8*
FLT3	c.1310-3T>C	p.?	rs2491231	*100*	*99.8*	*99.9*	*99.9*
KDR	c.798+54G>A	p.?	rs7692791	*99.7*	*99.6*	*99.9*	*100*
MET	c.3029C>T	p.T1010I	COSM707	*49.9*	*49.3*	*54.7*	*52.0*
MPL	c.1565+27G>A	p.?	-				*5.35*
PDGFRA	c.1701A>G	p.P567P	rs1873778	*99.9*	*99.6*	*99.9*	*99.9*
PDGFRA	c.2472C>T	p.V824V	COSM22413	*47.3*	*42.4*	*50.9*	*47.3*
RET	c.2307G>T	p.L769L	rs1800861	*52.5*	*44.2*	*52.3*	*44.9*
STK11	c.465-51T>C	p.?	rs2075606	*52.2*	*45.2*	*60.5*	*42.6*
TP53	**c.711G>A**	**p.M237I**	**COSM10834**		*5.89*		

pt. #407								
**GENE**	**Nucleotide variant**	**Amino acid mutation**	**COSMIC/dbSNP ID number**	**gDNA**	**FFPE**	**CTC M+/E+**	**CTC M+/E-**	**CTC M-/E-**
ALK	c.3541C>T	p.R181C	rs56315533				6.65	
APC	c.4479G>A	p.T1493T	COSM3760869			20.37		17.56
CSF1R	c.*1841TG>GA	p.?	rs386693509	96.67	96.61	98.25	99.00	100
EGFR	c.2361G>A	p.Q787Q	COSM1451600	100	99.58	100	99.00	100
EGFR	c.1750C>A	p.H584N	-				6.06	
EGFR	c.356T>C	p.L119S	-				6.50	
ERBB4	c.1786G>A	p.G596S	-				8.25	
EZH2	c.1934A>G	p.E645G	-			7.00		
FBXW7	c.1412A>G	p.E471G	COSM305935			7.74		
FGFR3	c.1953G>A	p.T651T	rs7688609	99,48	100	99.66	99,86	99.88
FLT3	c.2053+23A>G	p.?	rs75580865	54.81	64.65	59.23	41.65	74.15
FLT3	c.1310-3T>C	p.?	rs2491231	44.48	55.99	52.20	44.15	58.35
FLT3	c.2531T>C	p.V844A	-				5.26	
HRAS	c.81T>C	p.H27H	COSM249860	44.86	53.70	36.96	48.66	52.23
JAK3	c.420+18G>A	p.?	-			8.01		
JAK3	c.1737G>A	p.V579V	-				6.90	
KDR	c.798+54G>A	p.?	rs7692791	37.68	38.00	29.17	37.31	50.00
KDR	c.3423C>A	p.D1141E	-				9.65	
KDR	c.4004A>G	p.Q1335R	-				17.26	
KIT	**c.1638A>G**	**p.K546K**	**COSM21983**	50.22	49.72	59.93	44.90	45.19
KIT	c.1540+8T>C	p.?	-				5.30	
KIT	c.1994C>A	p.P665H	-				5.35	
KRAS	**c.172A>G**	**p.T58A**	**-**				8.20	
NRAS	c.130G>A	p.V44M	-			5.24		
NRAS	c.192C>T	p.Y64Y	-			5.47		
PDGFRA	c.1701A>G	p.P567P	rs1873778	100	99.93	100	99.84	100
PIK3CA	**c.3140A>T**	**p.H1047L**	**COSM776**			5.97		
PTEN	**c.1001A>G**	**p.N334S**	**COSM6948036**				6.26	
PTEN	**c.511C>T**	**p.Gln171Ter**	**COSM5149**				5.27	nc
PTPN11	c.1551G>A	p.A517A	COSM6135785				6.30	
RET	c.2307G>T	p.L769L	rs1800861	100	100	95.63	100	100
RET	c.2712C>G	p.S904S	rs1800836	48.11	47.09	nc	46.74	nc
RET	c.2285-8C>T	p.?	-				7.45	
SMAD4	c.1408C>T	p.P470S	-			5.85		
SMAD4	c.955+58C>T	p.?	rs948588	53.74	65.22	51.61	53.83	50.84
SMAD4	c.954T>C	p.P318P	rs773615487	52.79	65.56	49.25	54.15	43.58
SMAD4	c.1197C>T	p.V399V	-				6.86	
SMARCB1	c.573G>A	p.L191L	COSM6506621				7.60	
STK11	c.465-51T>C	p.?	rs2075606	45.31	69.69	41.94	57.26	nc
STK11	c.91T>C	p.S31P	-			6.63		
TP53	**c.388C>A**	**p.L130I**	**IARCTP53**				6.50	
TP53	c.215C>G	p.P72R	COSM250061	45.75	67.35	50	60.90	50.0
TP53	c.376-35T>C	p.?	-				7.43	
TP53	c.615T>C	p.Y205Y	rs786202222			6.4		

pt. #431						
**GENE**	**Nucleotide variant**	**Amino acid mutation**	**COSMIC/dbSNP ID number**	**gDNA**	**CTC M+/E-**	**CTC M-/E-**
APC	c.4479G>A	p.T1493T	COSM3760869	*100*	*99.95*	*80.86*
ATM	**c.5415G>A**	**p.W1805***	**COSM4551820**			*5.85*
ATM	c.8108A>G	p.D2703G	-			*5.95*
BRAF	**c.1800G>A**	**p.V600V**	**COSM249890**			*5.40*
CSF1R	c.*1841TG>GA	p.?	rs386693509	*100*	*100*	*91.26*
EGFR	c.2361G>A	p.Q787Q	COSM1451600	*56.22*	*51.54*	*57.62*
EGFR	c.2283+25G>A	p.?	COSM24212			*8.30*
EGFR	c.2266A>G	p.N756D	-			*8.33*
ERBB2	c.2621A>G	p.Glu874Gly	-			*5.73*
ERBB4	c.421+58A>G	p.?	rs839541		*7.06*	
FBXW7	c.796A>G	p.Lys266Glu	-			*5.80*
FGFR3	**c.2115G>A**	**p.K705K**	**COSM6282664**			*5.06*
FGFR3	c.1953G>A	p.T651T	rs7688609	*100*	*99.86*	*99.52*
FLT3	c.1310-3T>C	p.?	rs2491231	*50.00*	*49.30*	*59.60*
HRAS	c.81T>C	p.H27H	COSM249860	*43.22*	*55.26*	*58.5*
KDR	c.798+54G>A	p.?	rs7692791	*37.60*	*39.03*	*39.20*
KIT	**c.1638A>G**	**p.K546K**	**COSM21983**			*14.90*
KIT	c.2532T>C	p.C844C	-		*5.90*	
PDGFRA	c.1701A>G	p.P567P	rs1873778	*99.69*	*100*	*99.85*
PIK3CA	c.352+40A>G	p.?	rs3729674	*48.24*	*35.56*	*36.91*
PIK3CA	c.2765T>A	p.M922L	-		*7.06*	
PTEN	c.989A>G	p.K330R	-			*5.05*
RET	c.2307G>T	p.L769L	rs1800861	*100*	*90.53*	*95.53*
RET	c.2712C>G	p.S904S	rs1800836			*30.37*
RET	**c.2691A>G**	**p.R897R**	**COSM3723341**			*5.74*
RET	c.2289C>T	p.N763N	COSM918121			*6.09*
SMAD4	c.955+58C>T	p.?	rs948588	*51.33*	*48.60*	*44.76*
SMO	c.669A>C	p.T229T	ExAC		*5.90*	
SMO	c.642C>G	p.G214G	-		*5.75*	
SMO	c.675T>C	p.A225A	-		*5.60*	
SMO	c.600A>C	p.T200T	-		*5.95*	
SMO	c.576G>A	p.Q192Q	-		*5.85*	
SMO	c.573C>T	p.G191G	-		*5.95*	
STK11	c.465-51T>C	p.?	rs2075606	*53,04*	*38.79*	*53.48*
STK11	**c.980A>G**	**p.D327G**	**rs864622638**			*5.15*
TP53	c.215C>G	p.P72R	COSM250061	*97.81*	*99.22*	*99.38*

pt. #454							
**GENE**	**Nucleotide variant**	**Amino acid mutation**	**COSMIC/dbSNP ID number**	**gDNA**	**FFPE**	**CTC M+/E-**	**CTC M-/E-**
APC	c.4479G>A	p.T1493T	COSM3760869	*46.02*	*51.21*	*48.82*	*55.04*
CSF1R	c.954G>A	p.G318G	-				*5.70*
CSF1R	c.*1841TG>GA	p.?	rs386693509	*100*	*99.83*	*95.56*	*100*
EGFR	c.2361G>A	p.Q787Q	COSM1451600	*51.39*	*40.65*	*59.59*	*55.89*
FGFR3	c.1953G>A	p.T651T	rs7688609	*100*	*100*	*99.68*	*99.03*
FLT3	c.1310-3T>C	p.?	rs2491231	*100*	*99.95*	*99.95*	*99.90*
IDH1	c.315C>T	p.G105G	rs11554137	*53.43*	*50.03*	*49.95*	*43.65*
KDR	c.798+54G>A	p.?	rs7692791	*100*	*99.83*	*100*	*91.28*
PDGFRA	c.1701A>G	p.P567P	rs1873778	*100*	*99.85*	*100*	*100*
PDGFRA	c.2472C>T	p.V824V	COSM22413	*99.46*	*99.30*	*98.55*	*95.37*
RET	c.2307G>T	p.L769L	rs1800861	*99.49*	*100*	*100*	*99.85*
RET	c.2712C>G	p.S904S	rs1800836				*7.68*
STK11	c.465-51T>C	p.?	rs2075606	*54.92*	*53.05*	*49.14*	*52.45*
TP53	**c.388C>A**	**p.L130I**	**IARCTP53**			*7,03*	
TP53	c.215C>G	p.P72R	COSM250061	*99.20*	*97.78*	*99.09*	*99.11*

pt. #458							
**GENE**	**Nucleotide variant**	**Amino acid mutation**	**COSMIC/dbSNP ID number**	**gDNA**	**FFPE**	**CTC M+/E-**	**CTC M-/E-**
APC	c.4479G>A	p.T1493T	COSM3760869	*50.51*	*67.64*	*54,06*	*50.00*
CSF1R	c.*1841TG>GA	p.?	rs386693509	*100*	*100*	*100*	*84.29*
EGFR	c.2361G>A	p.Q787Q	COSM1451600	*100*	*99.62*	*100*	*nc*
FGFR3	c.1953G>A	p.T651T	rs7688609	*50.00*	*99.82*	*99.43*	*100*
FLT3	c.1310-3T>C	p.?	rs2491231	*50.00*	*73.59*	*53.98*	*44.80*
HRAS	c.81T>C	p.H27H	COSM249860	*42.29*	*37.46*	*43.59*	*65.43*
KIT	**c.1638A>G**	**p.K546K**	**COSM21983**				*5.52*
PDGFRA	c.1701A>G	p.P567P	rs1873778	*100*	*99.38*	*99.74*	*100*
PIK3CA	c.352+40A>G	p.?	rs3729674	*58.08*	*35.52*	*48.41*	*35.71*
RET	c.2307G>T	p.L769L	rs1800861	*49.78*	*48.00*	*45.41*	*55.74*
TP53	c.215C>G	p.P72R	COSM250061	*98.39*	*97.52*	*97.86*	*nc*
TP53	**c.817C>T**	**p.R273C**	**rs121913343**		*40.86*		

pt. #471							
**GENE**	**Nucleotide variant**	**Amino acid mutation**	**COSMIC/dbSNP ID number**	**gDNA**	**CTC M+/E+**	**CTC M+/E-**	**CTC M-/E-**
ALK	c.3559C>T	p.S1186S	-		20.59		
APC	c.4479G>A	p.T1493T	COSM3760869	49.21	50.46	44.00	50.75
ATM	c.1065+4C>T	p.?	-		6.72		
ATM	c.5226C>T	p.A1742A	rs750881004		10.57		
CDKN2A	**c.241C>T**	**p.P81S**	**COSM48299**		5.26		
CSF1R	c.941C>T	p.T314I	COSM4513528			6.95	
CSF1R	c.*1841TG>GA	p.?	rs386693509	100	100	100	99.12
CTNNB1	**c.136C>T**	**p.L46L**	**COSM5988491**		13.33		
EGFR	c.2361G>A	p.Q787Q	COSM1451600	45.06	60.21	44.58	47.62
ERBB4	c.421+58A>G	p.?	rs839541	100	99.30	100	91.86
ERBB4	c.2759C>T	p.P920L	-		10.56		
FGFR1	c.844T>C	p.S282P	-			7.40	
FGFR3	c.1953G>A	p.T651T	rs7688609	100	100	100	100
FGFR3	c.1959+22G>A	p.?	rs3135898	62.70	48.35	45.96	51.61
FGFR3	c.1959+21C>T	p.?	-	37.00	36.81	37.27	35.26
FGFR3	c.740-8C>T	p.?	rs886038257		13.25		
FLT3	c.1310-3T>C	p.?	rs2491231	99.79	99.91	99.95	84.82
HNF1A	c.795C>T	p.Y265Y	-		7.99		
HRAS	c.81T>C	p.H27H	COSM249860	50.96	45.59	56.09	41.35
KDR	c.798+54G>A	p.?	rs7692791	47.00	35.00	21.35	32.23
KDR	c.3849-16G>A	p.?	-				5.60
KDR	**c.2959G>T**	**p.E987Ter**	**-**		5.13		
KIT	**c.1621A>C**	**p.M541L**	**COSM28026**	48.22	63.31	37.81	43.87
KIT	c.1899A>G	p.E633E	-		10.44		
MPL	c.1549T>C	p.F517L	-			8.91	
NOTCH1	c.5020T>C	p.S1674P	COSM36230				5.63
NPM1	c.847-5T>C	p.?	rs191111314	50.37	50.00	44.21	48.11
PDGFRA	c.1701A>G	p.P567P	rs1873778	100	100	100	99.72
PIK3CA	c.1173A>G	p.I391M	rs2230461	52.71	47.22	53.05	51.29
PIK3CA	c.352+40A>G	p.?	rs3729674	48.69	62.38	55.56	48.88
PTEN	**c.794T>C**	**p.L265P**	**rs1335033900**				5.54
PTPN11	c.1571C>T	p.T524I	-		6.27		
PTPN11	c.1527A>G	p.A509A	-		6.34		
RB1	c.969A>G	p.E323E	-		7.11		
RET	c.2307G>T	p.L769L	rs1800861	51.66	47.10	46.94	48.50
RET	c.2712C>G	p.S904S	rs1800836	47.80	44.59	59.49	37.57
SMAD4	c.955+58C>T	p.?	rs948588		10.57		6.37
SMARCB1	c.1119-41G>A	p.?	COSM1090	49.11	61.54	47.58	45.13
STK11	c.465-51T>C	p.?	rs2075606	52.31	45.35	42.14	46.30
TP53	**c.388C>A**	**p.L130I**	**IARCTP53**		5.73		5.41
TP53	c.215C>G	p.P72R	COSM250061	99.35	97.78	97.04	96.37
TP53	**c.713G>A**	**p.C238Y**	**COSM11059**		6.65		
